# Genomic and functional adaptations in the guanylate-binding protein GBP5 highlight specificities of bat antiviral innate immunity

**DOI:** 10.1371/journal.pbio.3003760

**Published:** 2026-04-21

**Authors:** Amandine Le Corf, Sarah Maesen, Amandine Chantharath, Clara Loyer, Juan Manuel Vazquez, M. Elise Lauterbur, Veronika Krchlikova, Lucas Sareoua, Genavieve Gray-Sandoval, Andrea Cimarelli, Carine Rey, Peter H. Sudmant, David Enard, Lucie Etienne

**Affiliations:** 1 CIRI, Centre International de Recherche en Infectiologie, Inserm U1111, CNRS UMR5308, Ecole Normale Superieure de Lyon, Universite Claude Bernard Lyon 1, HCL, Univ Lyon, Lyon, France; 2 Department of Integrative Biology, University of California, Berkeley, California, United States of America; 3 Department of Biology, University of Vermont, Burlington, Vermont, United States of America; 4 Department of Ecology and Evolutionary Biology, University of Arizona, Tucson, Arizona, United States of America; Fred Hutchinson Cancer Research Center, UNITED STATES OF AMERICA

## Abstract

Bats are asymptomatic reservoirs of several zoonotic viruses. This may result from long-term co-evolution between viruses and bats, that have led to host adaptations contributing to an effective balance between strong antiviral responses with innate immune tolerance. To better understand these virus-host interactions, we combined comparative transcriptomics, phylogenomics and functional assays to characterize the evolution of bat innate immune antiviral factors. First, we stimulated the type I interferon immune pathway in *Myotis yumanensis* primary cells and identified guanylate-binding protein 5 (GBP5) as the most differentially expressed interferon-stimulated gene (ISG). Phylogenomic analyses showed that bat GBP5 has been under strong episodic positive selection, with numerous rapidly evolving sites and species-specific gene duplications, suggesting past evolutionary arms races. Functional tests on GBP5 orthologs from 10 bat species covering the >60 million years of Chiroptera evolution revealed species- and virus-specific restrictions against RNA viruses (retrovirus HIV, and rhabdoviruses European bat lyssavirus and VSV), which are typical signatures of adaptations to past viral epidemics. Interestingly, we also observed a lineage-specific loss of the GBP5 prenylation motif in the common ancestor of *Pipistrellus* and *Eptesicus* bats. Importantly, resurrection of the prenylation motif in *Eptesicus fuscus* GBP5 in corresponding bat cells was associated with different GBP5 subcellular localization and loss of anti-rhabdoviral functions, suggesting specific adaptation to ancient viral epidemics ~22 million years ago. Altogether, our results highlight adaptations that contribute to bat specific immunity and provide insights into the functional evolution of antiviral effector GBP5.

## Abstract 2

Bats harbor many zoonotic viruses yet remain asymptomatic, and the evolutionary basis of their antiviral tolerance remains unclear. This study shows that bat GBP5 evolved under positive selection, driving virus-specific restriction and lineage-specific loss of a prenylation motif that reshaped antiviral function.

## Introduction

The mammalian antiviral innate immune system has been shaped by ancient conflicts with pathogenic viruses, which must constantly adapt to evade or antagonize host antiviral responses [1,2]. These past viral epidemics exerted selective pressures favoring genomic adaptations that naturally arose in the host and were a benefit against pathogenic viruses, either capable of inhibiting virus replication or limiting the development of pathogenesis. Over evolutionary time, host-virus arms races have left signatures of adaptations in host genomes, such as site-specific positive selection by nonsynonymous mutations, gene duplications or indels [1]. Past host adaptations may provide modern immune specificities against existing viruses. These immune characteristics may be important in the context of cross-species transmissions and in shaping reservoir host species, which asymptomatically host viruses.

Among mammals, bats are reservoirs of a wide range of viruses, some of which are pathogenic to other species and have been the source of zoonotic outbreaks in humans [3]. With over 1,400 species spread across all continents and over 60 million years of divergence, bats have a long and diverse evolutionary history with viral infections. This long co-evolution has contributed to shaping their immune system and led to specific adaptations, which may participate in their efficient control of viral infections. Bats have altered expression and function of inflammasome components and other innate immune sensors, including the cellular receptors NLRP3 [4], PYHIN [5], and AIM2. For example, the loss of inflammasome activation in some bat species may reduce the deleterious excess of inflammatory responses upon viral infections and may be protective. Despite this enhanced tolerance, their antiviral defenses appear strong and diversified. Several bat antiviral effectors, particularly from the interferon (IFN) innate immune pathway, present signatures of strong species-specific positive selection and gene duplications, which may be reminiscent of past host-pathogen arms races. This is for example the case for the *Guanylate-binding protein (GBP)* gene family, which encodes effectors against bacteria, parasite and viral infections [6], and has undergone gene duplications/losses and site-specific positive selection during Chiroptera evolution [7]. However, no functional study has characterized the potential drivers or consequences of GBP diversifications yet. In fact, amongst the thousands of mammalian ISGs, only few studies combined evolutionary and functional approaches in bats to formally reveal their adaptations to past viral epidemics. Studies on protein kinase R (PKR) [8], the Mx family of guanosine triphosphatases (e.g., Mx1) [9], interferon-induced transmembrane protein 3 (IFITM3) [10,11], or ISG15 [12] showed evidence of adaptive duplications, splice variants or site-specific adaptations in response to DNA and RNA viral infections. Yet, because of the lack of genomic and cellular tools, evolutionary and functional understanding on the antiviral innate immune response in bats remains poorly investigated.

We previously identified strong signals of positive selection in antiviral immune factors of the *Myotis* bat lineage [13]. Here, we therefore derived primary cells from three *Myotis yumanensis* individuals and performed a transcriptomic analysis of their IFN innate immune response. This allowed us to identify guanylate-binding protein 5 (GBP5) as the most differentially upregulated interferon-stimulated gene (ISG) in *Myotis yumanensis* primary cells.

Human GBP5 is an IFN-inducible antiviral factor that restricts a broad spectrum of intracellular pathogens [14,15], including viruses such as HIV-1 (Human immunodeficiency virus 1), SARS-CoV-2 (severe acute respiratory syndrome *coronavirus 2*), or bat lyssavirus EBLV-1 (European Bat LyssaVirus 1) [16–18]. GBP5 notably targets viral glycoprotein processing, leading to the release of new virions with immature glycoproteins, thereby reducing virions’ infectivity [19]. Although the exact mechanisms are still unknown and debated [19–21], this viral restriction appears dependent on human GBP5 localization at the *trans-*Golgi network (TGN), thanks to its prenylation at a CaaX motif in the C-terminal end of the protein [18]. We further reasoned that studying natural variations in this antiviral effector during mammalian evolution would help understand its functions.

We therefore combined phylogenomic and functional approaches including in bat cells to determine bat GBP5 adaptation and function against modern viruses. We found that bat GBP5 has been under strong episodic positive selection and experienced gene duplication, suggesting important past evolutionary arms races. Functional tests on GBP5 from a large panel of 10 bat species revealed species-specificity of restriction against viral particles bearing glycoproteins of diverse RNA viruses: HIV-1, VSV (vesicular stomatitis virus) and EBLV-1. Interestingly, we also found lineage-specific loss of the prenylation motif, correlating with intracellular and antiviral phenotypic variations. Resurrection of the ancestral GBP5 prenylation motif rescued the subcellular localization, but was detrimental against VSV-GFP infections. These results highlight adaptations, possibly driven by past rhabdoviral-like epidemics, that contributed to bat immunity and result in specific modern host-virus restrictions.

## Results

### Transcriptomics of primary cells derived from three *Myotis yumanensis* individuals after immune stimulation unveil GBP5 as a top ISG

**Fig 1:**
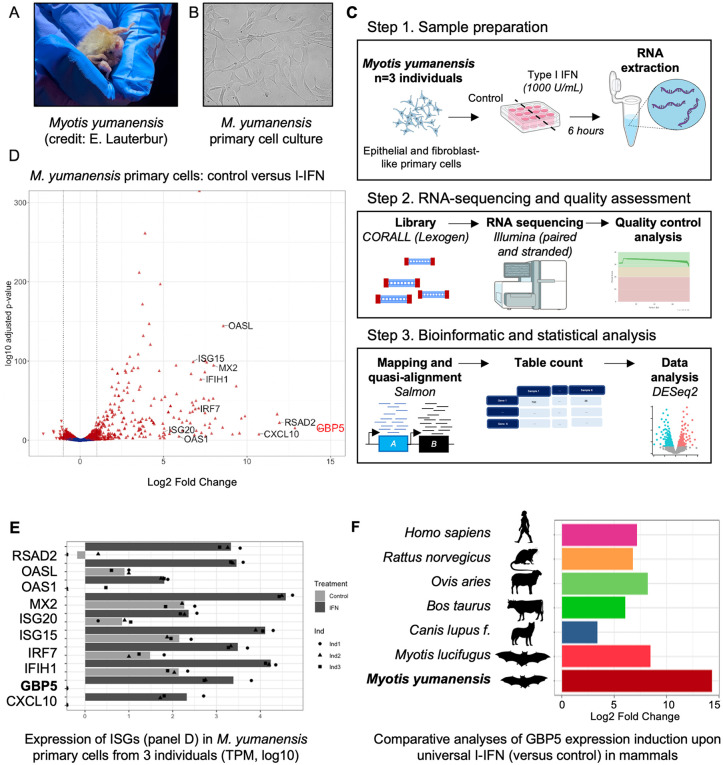
GBP5 is the most differently upregulated ISG in *Myotis yumanensis* primary cells upon type I interferon immune stimulation. **A,** Photo of a *Myotis yumanensis* bat at a sampling site (Credit: Elise Lauterbur). **B,** Primary cells derived from a biopsy wing punch. **C,** Schematic of the workflow from bat sampling to RNA sequencing analyses. Bat primary cell lines were similarly derived from three *M. yumanensis* individuals and treated with, or without, universal type I IFN to trigger ISG expression. Total RNA was extracted and sequenced, followed by analyses to identify differentially expressed genes between stimulated cells compared to control cells. **D,** Volcano plot representing the differential gene expression between untreated and IFN-stimulated cells from three *M. yumanensis* individuals. Genes significantly (*p* < 0.05) differentially expressed (DE) are colored in red. Several known ISGs are highlighted on the graphic. GBP5 is the most upregulated ISG. **E,** Expression of ISGs from panel D in *M. yumanensis* primary cells derived from three individuals in control (light gray) and I-IFN (dark gray) conditions. TPM, Transcripts per million counts (log10 scale; complete table in S1 Dataset). **F,** Comparative analysis of differential gene expression (Log2 Fold Change) of GBP5 upon universal type I IFN treatment *M. yumanensis* cells as compared to six other mammalian species cells in a similar experimental setup ([22]; details in Materials and methods). Pictos from https://www.phylopic.org/ and Illustrations from NIAID NIH BioArt Source (https://www.bioart.niaid.nih.gov/). The data underlying this Figure can be found in S1 and S2 Dataset. See Data availability for access to the code.

To better characterize cell-autonomous innate immunity in bats, we performed a transcriptomic approach in primary cells from three individuals of *Myotis yumanensis* under type I interferon (IFN) stimulation (Fig 1A–1C). We chose this species because it has genome-wide evidence of adaptations in response to viral infections and exceptional genome sequence and annotation [13]. Briefly, primary fibroblast-like cells were derived from wing punches of three *M. yumanensis* bat individuals in the US Southwest (see Materials and methods). Following previous studies on the IFN stimulation of bat cell lines [22–24], we stimulated the primary cells with, or without, universal type I IFN for 6 hours. We then isolated total cellular RNA for sequencing (RNAseq) and subsequent bioinformatic analyses. We used Salmon [25] to quantify *M. yumanensis* gene expression. The genome of *M. yumanensis* is one of the best annotated genomes of all mammals, with BUSCO scores of 94.5% and around 19,000 annotated protein-coding genes, leaving few unannotated regions [13]. This allowed extremely high quality of our RNAseq with one-to-one (human and *Myotis*) ortholog analysis. Through pairwise comparisons with DESeq2 [26], we identified genes that were significantly upregulated (*n* = 1,036) and downregulated (*n* = 859) by a log2 fold change (FC) ≥ 2 and with an adjusted *p* value < 0.05, and which we defined as significantly responding to type I-IFN (Fig 1D). Amongst the significantly upregulated genes, we found several innate immune factors, indicating that I-IFN treatment led to an efficient activation of the innate immune responses in these primary cells. Specifically, among the most differentially expressed genes, we identified some involved in the innate immune signaling pathways (e.g., IRF7, IFIH1), cytokines (e.g., CXCL10) and antiviral ISGs, such as ISG15, ISG20, OAS1, OASL, Mx2, or RSAD2/Viperin (Fig 1D, 1E; Fig 1E highlights counts per gene in each condition and per *M. yumanensis* individuals). Notably, GBP5 was the most differentially expressed ISG in *M. yumanensis* primary cells compared to the unstimulated control in which GBP5 is not expressed (Fig 1D, 1E; of note, the lower significance for GBP5 in Fig 1D was solely due to more variations in differential expression between cells derived from different *Myotis* individuals, which do not express any GBP5 at basal level, similarly to CXCL10 or RSAD2, as shown in Fig 1E), an interesting finding in light of the broad antiviral activities recently ascribed to this factor.

To decipher whether this was typical of mammalian GBP5 expression upon interferon stimulation, we performed a comparative analysis. We used publicly available results from a transcriptomic study defining the interferome of cells among mammalian species treated in similar conditions [22]. We found that GBP5 was also an ISG in six other mammalian species (Fig 1F). Yet, the upregulation of GBP5 transcripts in *Myotis yumanensis* primary cells seemed remarkably high compared to the others, including its closest related species with transcriptomic information, *Myotis lucifugus* (1.5-fold higher; Fig 1F).

### GBP5 has evolved under episodic positive selection during mammalian evolution

**Fig 2:**
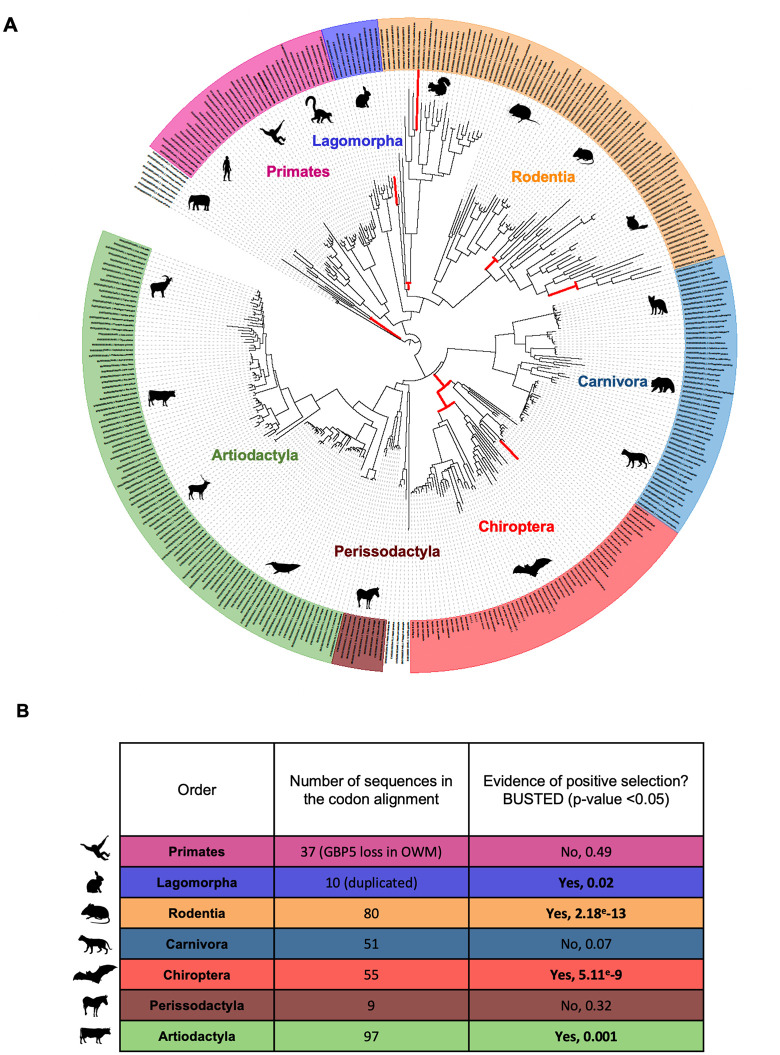
Mammalian GBP5 has evolved under episodic positive selection, with strong diversification in rodents and bats. **A,** Phylogenetic analysis of GBP5 across mammals. The phylogeny was built from a PRANK [28] codon alignment of 348 GBP5 homologous sequences. Maximum likelihood phylogenetic tree was built with IQ-TREE [29] with 1000-bootstrap replicates for statistical support (see Data availability). Branches under significant positive selection (*p*-value < 0.05) assigned by aBSREL are thickened and in red. The scale bar indicates the number of substitutions per site. **B,** Evidence of positive selection in GBP5 from several mammalian orders. Positive selection analyses were performed with BUSTED [30] using a PRANK codon alignment for each mammalian order. OWM, Old world monkeys. Species silhouettes are from https://www.phylopic.org. The data underlying this Figure can be found in S2 Dataset, the alignments for phylogenetic analyses are all openly available in FigShare Dataset (https://doi.org/10.6084/m9.figshare.26180785.v1, https://doi.org/10.6084/m9.figshare.26180764.v1, https://doi.org/10.6084/m9.figshare.26180803.v1, https://doi.org/10.6084/m9.figshare.26180761.v1, https://doi.org/10.6084/m9.figshare.26180767.v1, https://doi.org/10.6084/m9.figshare.26180776.v1, https://doi.org/10.6084/m9.figshare.26180794.v1, https://doi.org/10.6084/m9.figshare.26180797.v1).

Antiviral factors that are important in vivo have often evolved under recurrent positive selection, as a consequence of their engagement in molecular arms races with pathogenic viruses [2,27]. We therefore characterized the evolutionary history of GBP5 in mammals, using phylogenetic and positive selection analyses. First, we retrieved GBP5 homologous sequences in mammalian genomes from public databases using human GBP5 as a query (details in Materials and methods). We further obtained de novo sequences of bat GBP5s from closely related *Myotis* species that we sampled in the wild and from a cell line of *Eptesicus fuscus* (see Materials and methods). In total, we analyzed 348 nucleotide sequences of mammalian orthologous GBP5s from the main orders: Chiroptera (*n* = 55), Rodentia (*n* = 80), Primates (*n* = 37), Perissodactyla (*n* = 9), Artiodactyla (*n* = 97), Lagomorpha (*n* = 10), and Carnivora (*n* = 51), as well as other mammalian sequences that were used as outgroups: Proboscidea (*n* = 3), Pilosa (*n* = 1), Cingulata (*n* = 4), and Xenarthra (*n* = 1). We then codon-aligned the GBP5 coding sequences with PRANK [28] and performed phylogenetic analyses with IQ-TREE [29]. The GBP5 gene tree seemed to follow the accepted mammalian species tree (Fig 2A), with some exceptions due to gene duplications/losses. For example, we confirmed an ancient duplication event in Lagomorpha [31] and the loss of GBP5 in primate Old World Monkeys [32,33]. We also detected duplication events, indels and high variability in sequences during rodent and bat evolution (Fig 2A, See Data Availability for alignments and phylogenetic details). To identify whether, and when, episodic positive selection of GBP5 has occurred during mammalian evolution, we used a branch-specific method, adaptive Branch-Site Random Effects Likelihood (aBSREL) [34]. aBSREL tests whether a given lineage has undergone significant positive selection and estimates the ω ratio, which is the ratio of the nonsynonymous to synonymous substitution rates (dN/dS). We found evidence of episodic positive selection in GBP5 during mammalian evolution, in particular in rodents, primates, lagomorphs, and bats (Fig 2A). We further analyzed each mammalian order independently, using BUSTED [30] from HyPhy [35]. We confirmed that mammalian GBP5 has evolved under positive selection in several orders (Fig 2B), particularly during Rodentia and Chiroptera evolution, as well as in Artiodactyla and Lagomorpha.

### GBP5 is under strong diversifying evolution in bats at the genomic and genetic levels

**Fig 3:**
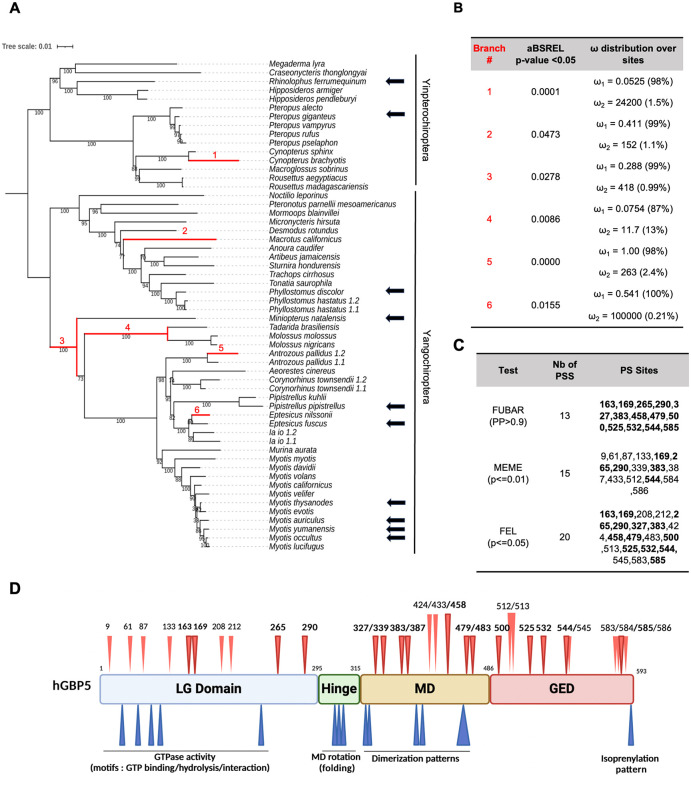
GBP5 has evolved under strong positive selection in bats. **A,** Phylogenetic and positive selection analyses of bat GBP5. Maximum likelihood phylogenetic tree of bat GBP5 was built with IQ-TREE and statistical support is from 1,000 bootstrap replicates (values are shown below branches). Branches under significant positive selection (*p*-value < 0.05) assigned by aBSREL are in red and the corresponding estimated values of ω are reported in panel B. The scale bar indicates the number of substitutions per site. The black arrows identify species with GBP5 genes that were functionally tested in this study. 1.1 and 1.2 identify GBP5 duplicates within a given bat species. **B,** Evidence of lineage-specific positive selection during bat GBP5 evolution. aBSREL identified at least six branches under significant positive selection. ω1 and ω2, estimation of ω in the rate class not allowing positive selection, and allowing positive selection (ω > 1), with % of sites in this class in parentheses, respectively. **C,** Sites under positive selection (PS) in bat GBP5. Site-specific positive selection analyses were performed using FUBAR [36], MEME [37], and FEL [38] from HYPHY/Datamonkey [35,39]. Only the sites above the indicated “statistically significant cut-off” are shown (PP, posterior probabilities for FUBAR; *p*-value for MEME and FEL). In bold are the sites identified by several methods. Nb of PSS, number of positively selected sites. “PS sites” numbering is according to the codon numbering in the PRANK codon alignment. **D,** Schematic representation of GBP5 with its functional domains and the herein identified sites under positive selection (red arrows at the top). LG domain, large GTPase domain. MD, Middle domain. GED, GTPase effector domain. The size of the domains is not to scale. Residues identified by at least two positive selection methods are highlighted in bold. In blue, sites or motifs involved in known functions in human GBP5. The bat phylogenetic tree from Fig 3A can be found in https://doi.org/10.6084/m9.figshare.30924512 and all results from positive selection analyses in https://doi.org/10.6084/m9.figshare.30924551.

Because we found evidence of positive selection in bat GBP5 at the gene-wide level, as well as pairwise-amino acid sequence divergence that could reach 20% within Chiroptera (S1 Fig), we performed comprehensive evolutionary analyses in this order. To identify when positive selection has occurred during bat evolution, we used aBSREL [34] from the Chiroptera-specific GBP5 alignment. We found that several bat lineages were the targets of intensive episodic positive selection, including the branch at the origin of the Microchiroptera suborder, as well as other internal and terminal lineages spread-out in the Yangochiroptera and Yinpterochiroptera clades (Fig 3A, 3B). This indicates differential selective pressure during bat evolution and may represent lineage-specific adaptations driven by past viral epidemics.

Beyond evidence of lineage-specific positive selection, we identified multiple, independent duplication events in bat GBP5, specifically in *Phyllostomus hastatus, Antrozous pallidus, Corynorhinus townsendii*, and *Ia io* (Fig 3A), which diverged a long time ago (Fig 3A). Despite all the duplication events being recent (i.e., only at terminal branches), we found important divergence between the copies (Figs 3A and S1).

Furthermore, we carried out several analyses to specifically identify the sites that have evolved under positive selection. Using three maximum likelihood methods from HyPhy (see Materials and methods), we found many positively selected residues in bat GBP5 (Fig 3C). Conservatively, considering only residues identified by at least two methods, we identified 16 positively selected sites—of note, if we consider sites identified by at least one method, we have 30 sites total. The rapidly evolving residues are found in all GBP5 protein domains, except for the hinge domain, which contains no positively selected sites (Fig 3D). Overall, we identified a very high concentration of sites under positive selection, as well as numerous indels (insertion/deletion) and lineage-specific early stop codons in the C-terminal part of bat GBP5.

Taken together, our transcriptomic and phylogenomic approaches (Figs 1–3) indicate that GBP5 could be an important innate immune factor engaged in a molecular conflict in bats. This suggests that GBP5 could play important and lineage-specific roles against various pathogens and that past epidemics may have shaped bat innate immunity.

### Natural variation in subcellular localization of bat GBP5

To test how past genetic diversification may have impacted GBP5 functions, we focused on 10 bat species (indicated by the black arrows in Fig 3A) that represent: (i) a large panel of orthologous GBP5 sequences encoding for maximum amino acid variations at the sites identified under positive selection (S2 Fig) and (ii) covering various bat families of different clades—some of which are very distant from each other while others are very close (e.g., *Myotis spp*.). This approach allowed us to functionally cover the evolution of bat GBP5 over a period of around 60 million years, at both ancient and more recent times.

In humans, the localization of GBP5 at the TGN is absolutely required for its antiviral activities [16–18]. Its localization is driven by a prenylation sequence present at the C-terminal end of the protein (CaaX motif) that anchors GBP5 at the TGN membrane. Synthetic mutation in this domain prevents both TGN localization and antiviral activities (*Homo sapiens* GBP5-C583A) [16,40].

**Fig 4:**
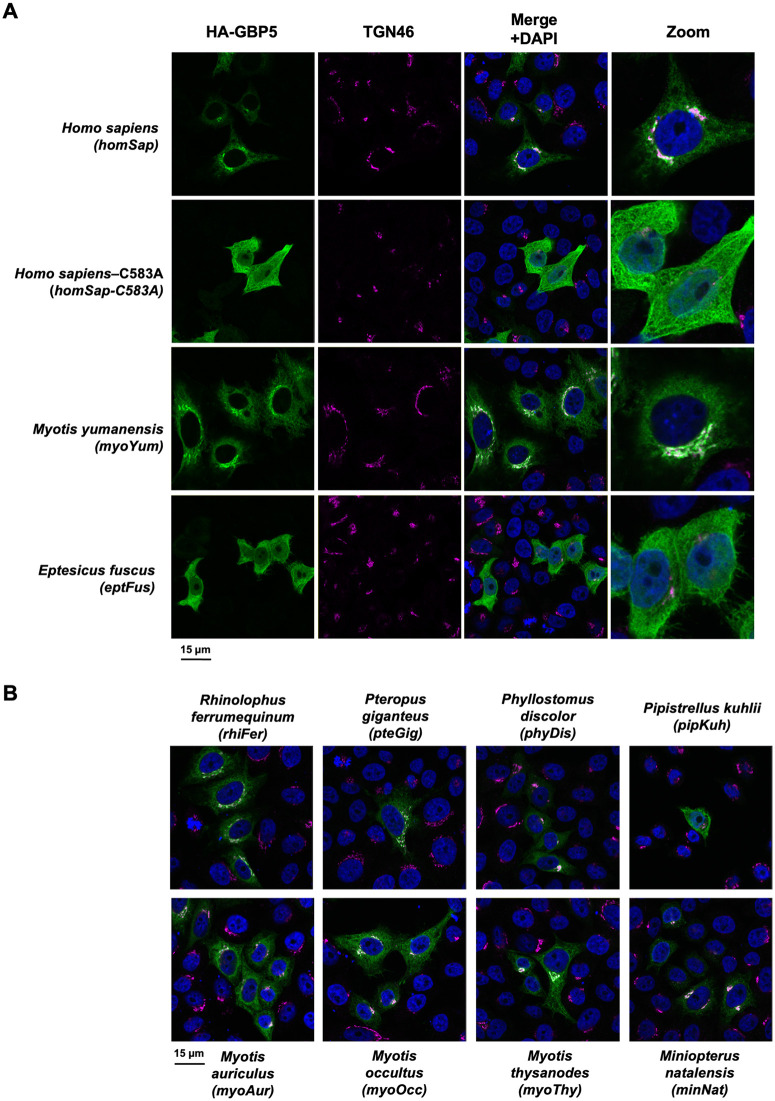
Natural variation in subcellular localization of bat GBP5s. TZM-bl cells were transfected with a plasmid coding for indicated HA-GBP5 species proteins: 10 bat orthologs, and 2 human GBP5s: wt and mutant C583A. Two days post-transfection, GBP5 localization was analyzed by confocal fluorescence microscopy with the indicated marker. Nuclei and *trans-*Golgi-network (TGN) were stained with DAPI and anti-TGN46, respectively. **A,** All the channels and a zoom are shown for *Homo sapiens*, the mutant *Homo sapiens-C583A, Myotis yumanensis* and *Eptesicus fuscus*. **B,** Only the merge is shown for the remaining bat species. The complete panel is shown in S3 Fig. The pictures present representative results observed in 3 independent experiments. Scale bar indicates 15 μm.

To study the subcellular localization of bat GBP5, we cloned the 10 selected orthologous bat GBP5s (Fig 3A) with an HA tag in a eukaryotic expression vector, and compared them to human GBP5 (wt), a prenylation-defective mutant (human GBP5-C583A) and a control vector (i.e., not expressing any GBP5). We ectopically expressed GBP5 in TZM-bl cells and imaged the cells 48 hours later by confocal immunofluorescence microscopy. We found colocalization of GBP5 with the TGN46 marker for 8 out of 10 bat species (Fig 4), showing GBP5 localization to the TGN similarly as human GBP5. Surprisingly, two closely related bat species *Eptesicus fuscus* and *Pipistrellus kuhlii* exhibited a diffused subcellular distribution in the cell, similarly to what we observed for the *Homo sapiens*-C583A mutant (Fig 4). Thus, bat GBP5s present natural variations in their subcellular localization, from TGN to cellular diffusion.

### Bat GBP5s restrict the retrovirus HIV-1 in a species-specific manner

The antiviral functions of human GBP5 have been particularly studied in the context of HIV-1 infection [16,18], where it was shown to target HIV-1 envelope glycoproteins, reducing the intrinsic infectivity of neosynthesized virions. Specifically, GBP5 alters the cleavage of the envelope precursor gp160 in the TGN, preventing maturation into gp120 and gp41 proteins, thereby leading to the production of immature viral particles. However, the precise mechanisms and whether additional inhibitory effects exist remain unclear. Recent reports showed that human GBP5 specifically alters HIV-1 gp120 glycosylation, which can be experimentally witnessed by a typical electrophoretic mobility shift of viral glycoproteins [16,18]. Nonetheless, the anti-retroviral effect of human GBP5 is strictly dependent on its TGN localization. We therefore assessed the antiviral activity of bat GBP5s, using HIV-1 replication.

**Fig 5:**
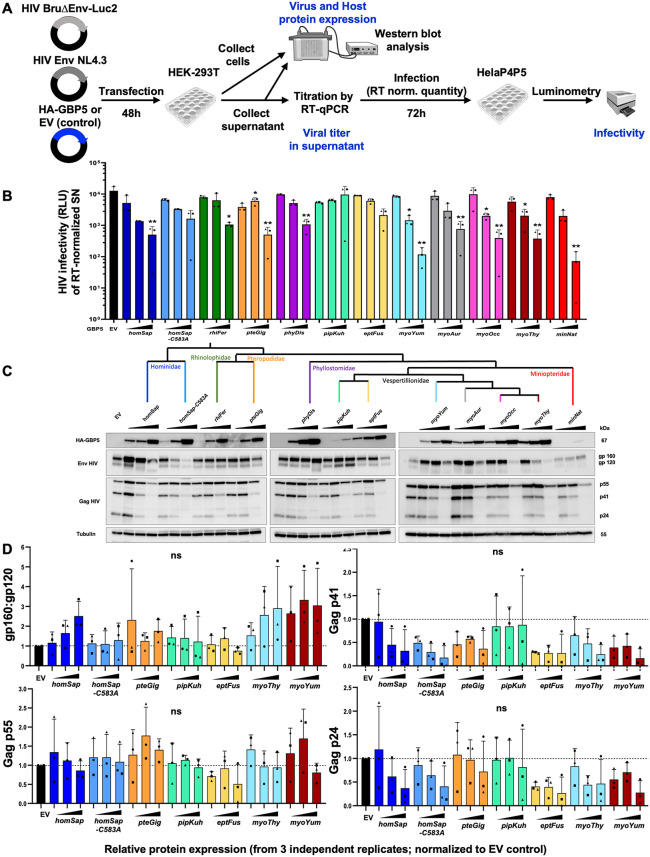
Species-specificity in bat GBP5 restriction of retrovirus (HIV-1). **A,** Experimental setup. Briefly, HEK-293T cells were transfected with plasmids coding for HA-GBP5 or the control (EV “empty vector” expressing E2-Crimson), and for HIV-1 LAI genome and Luciferase reporter (Bru∂EnvLuc2 vector), NL4.3 Envelope and Rev. 48 hour post-transfection, cells were harvested for western blot analysis, and supernatant for western blot analysis after ultracentrifugation, titration of virus by RT-qPCR (RT activity), and infection of the RT-normalized viruses in HeLaP4P5 cells. Infection was quantified 72 hour later by luminescence from the viral-encoded Luciferase reporter. **B,** HIV-1 intrinsic infectivity (RLU, Relative light units) normalized to RT activity in the supernatant, in the context of control (EV, encoding solely E2-crimson) or a dose of HA-GBP5 (1, 2, or 4 µg; total DNA identical across conditions). The corresponding species of GBP5 is shown (name follows the UCSC nomenclature, three letters from genus followed by three letters from species). Results from three independent experiments, bars correspond to SD. Statistics vs. the corresponding control EV condition, by one-way ANOVA, Dunnett’s test: *, *p* value < 0.05, **, *p* value < 0.01. **C** and **D**, Corresponding western blot analyses of HA-GBP5, HIV-1 Env, and HIV-1 Gag, and Tubulin from the total cell lysates of the HIV-1 producer cells in the context of a GBP5 dose (constant total transfected DNA). The cladogram at the top shows the phylogenetic relationships of the tested species. The RT activity titers and a western blot of purified supernatants are in S4 Fig. Panel C shows one representative experiment and panel D the quantification of western blots from three independent experiments (Tubulin is in S5A Fig). Illustrations from NIAID NIH BioArt Source (https://www.bioart.niaid.nih.gov/). The data underlying this Figure can be found in S2 Dataset.

HEK-293T cells were cotransfected with increasing doses of GBP5 or control vector (Empty vector, EV, encoding E2-Crimson only), as well as a plasmid encoding the HIV-1 envelope (Env NL4.3) and an HIV-1 genome plasmid expressing all other HIV-1 proteins along a luciferase reporter (Fig 5A for the experimental setup). Total DNA transfected was held constant across conditions. Two days later, we quantified viral particles in the supernatant, by measuring HIV-1 reverse transcriptase (RT) activity with RT-qPCR (S4A Fig). We noted that only high doses of GBP5 affected HIV-1 RT activity in the supernatant (up to 10-fold reduction for some species, S4A Fig and S1 Table). To determine if, as human GBP5, bat GBP5s influenced the intrinsic infectivity of the neosynthesized viral particles, we infected HeLa P4P5 cells (i.e., HeLa cells stably expressing the HIV-1 receptors and co-receptors) with the same amount of HIV-1 virions: equivalent to 30 mU RT. In this experimental setup, we measure the effect of GBP5 on HIV infectivity independently of the possible effect on the amount of produced viral particles (Fig 5A). Three days post-infection, we found that several bat GBP5s significantly inhibited the virion intrinsic infectivity of HIV-1 in a dose-dependent manner, similarly to human GBP5 (Fig 5B and S1 Table). As examples, GBP5 from *Pteropus giganteus (pteGig), Miniopterus natalentis (minNat), Phyllostomus discolor (phyDis)*, and *Myotis spp*. (myoXxx) significantly restricted HIV-1 intrinsic infectivity, with up to 1-log restriction compared to the control condition (EV). Interestingly, the GBP5s from three bat species, which were well expressed in the cells (Fig 5C), exhibited a distinct behavior: *Rhinolophus ferrumequinum (rhiFer)* GBP5 showed only mild HIV-1 restriction, and *Pipistrellus kuhlii (pipKuh)* and *Eptesicus fuscus (eptFus)* GBP5s did not restrict intrinsic infectivity, similarly to the human GBP5-C583A mutant (Fig 5B).

To further characterize viral proteins in the presence of GBP5s, we analyzed and quantified their expressions by western blot in cell lysates from the three independent experiments (Figs 5C, 5D, and S5A). Overall, we did not find any statistically-significant differences in viral protein expression levels; yet, in the producer cells, there was a trend for Gag p41 and p24 decreased expression by some GBP5s, as compared to Gag p55 (Figs 5C, 5D, and S4B). Of note, we did not observe any variation in Tubulin expression in these assays, in which we had collected total cell lysates and run the same volumes (S5A Fig). We also probed virus supernatants from two experiments (S4B Fig shows one), which suggested possible lower expression of the mature gp120 and increased expression of the immature gp160 precursor in restrictive GBP5s. In the cell, this seemed concomitant with a change in the electrophoretic mobility of Env, as well as mild, albeit not statistically-significant, changes in the gp160:gp120 ratios (Fig 5C, 5D), as previously reported for human GBP5 [19]. Yet, we found a case in which GBP5 from *Pteropus giganteus* (pteGig) strongly restricted HIV-1 intrinsic infectivity (Fig 5B) without any evidence of electrophoretic mobility of Env (Fig 5C–5E). It is therefore possible that, beyond previously described phenotypes on glycoproteins and independently of its prenylation, GBP5 may have additional antiviral functions, which remain to be investigated. Finally, as control, we performed cell viability assays in the context of HIV and GBP5 exogenous expressions and found no evidence of general cytotoxicity (S5B Fig).

Overall, we revealed that bat GBP5s are antiviral factors, restricting HIV-1 replication by inhibiting viral intrinsic infectivity, but that this is species-specific, suggesting that bat GBP5 diversification is the result of virus-host arms races.

### Virus-specificity and increased antiviral breadth in bat GBP5s

To determine the breadth of the bat GBP5 antiviral restriction, we tested their effect on the infectivity of viral particles bearing glycoproteins from two other RNA viruses: VSV and EBLV-1, which are independent of furin cleavage. Previous studies showed that human GBP5 inhibits the infectivity of retrovirus (HIV) pseudotyped with the glycoprotein of EBVL-1 (isolated from *Eptesicus serotinus* bats) but not with that of VSV [18]. Yet, a recent report by Veler *and colleagues* 2024 found that human GBP5 does restrict HIV:VSVg infectivity, strongly suggesting that GBP5 has an antiviral activity independent of glycoprotein cleavage by the furin [19].

**Fig 6:**
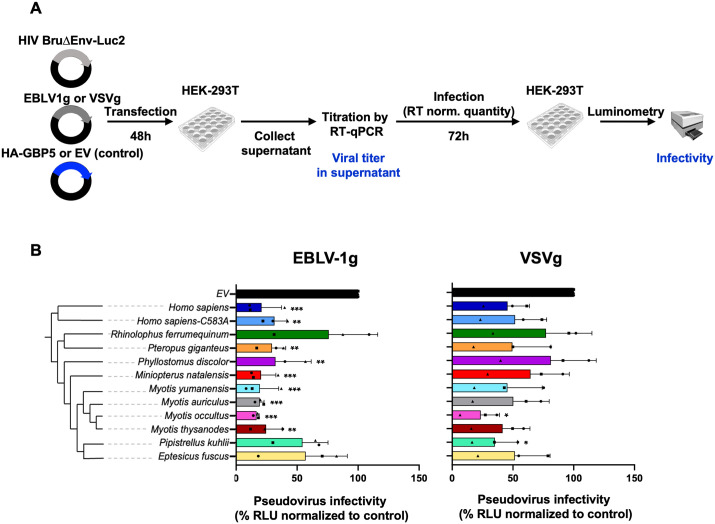
Bat GBP5 restriction is both species- and virus-specific. **A,** Experimental setup similar to Fig 5A but with viral pseudotyping with VSVg (VSV condition) or EBLV-1 envelope (EBLV-1 condition). **B,** Infectivity of RT-normalized pseudotyped-viruses produced in the presence of GBP5, normalized to the condition without GBP5 (EV, Empty vector E2-Crimson control) at 100%. The cladogram on the left shows the phylogenetic relationships of the tested GBP5 species. RLU, Relative light units. Results from three independent experiments, bars correspond to SD. Statistics vs. the corresponding control condition, One-way ANOVA, Dunnett’s test: *, *p* value < 0.05, **, *p* value < 0.01, ***, *p* value < 0,001. Illustrations from NIAID NIH BioArt Source (https://www.bioart.niaid.nih.gov/). The data underlying this Figure can be found in S2 Dataset.

Here, HEK-293T cells were cotransfected with 2 µg GBP5 or the control vector, and plasmids encoding HIVΔenv luciferase reporter viruses pseudotyped with EBLV-1g or VSVg (HIV:EBLV-1g or HIV:VSVg, respectively). We quantified RT activity from the supernatant, as previously described, and measured the virions’ infectivity in new HEK-293T target cells (Fig 6A for the experimental setup). First, for both pseudoviruses, we similarly observed a decrease in RT activity in the supernatant for all GBP5s (S6 Fig). Second, and after normalization of virion amounts (RT activity), we found that bat GBP5s reduced the infectivity of HIV:EBLV-1g, but in a species-specific manner in the magnitude of restriction (Fig 6B). GBP5 from *Pteropus giganteus, Phyllostomus discolor, Miniopterus natalensis*, and *Myotis spp.* significantly reduced HIV:EBLV-1g infectivity by 70%–80%. Interestingly, the same inhibitory effect was observed with the human GBP5 and GBP5-C583A mutant, suggesting a restriction mechanism independent of GBP5 isoprenylation at the CaaX motif. In *Rhinolophus ferrumequinum*, *Pipistrellus kuhlii*, and *Eptesicus fuscus*, the restriction was milder in the range of 35−45%. By comparison with HIV:HIV-1-Env restriction, these are the same species with the least impacting antiviral effect. This suggests that genetic variations in these bat GBP5s are probably located at common interfaces required for restriction of EBLV-1g and HIV Env mediated infectivity (i.e., common determinants). Third, we found that most bat GBP5s, as human GBP5, had a mild inhibitory effect on HIV:VSVg infectivity. Yet, GBP5 from two *Vespertilionidae* bat species, *Myotis occultus*, and *Pipistrellus kuhlii*, significantly restricted HIV:VSVg infectivity (up to 80% inhibition) (Fig 6B). In the latter case, it was particularly unexpected given the lack of HIV infectivity restriction (Fig 5) by *Pipistrellus kuhlii* GBP5. It therefore supports that GBP5 antiviral functions can also be virus-specific. It further suggests that specific genetic innovations in GBP5 from some *Vespertilionidae* enabled a gain in antiviral activity, contributing to specificities unique to them. Altogether, these results show that the antiviral restriction of bat GBP5 is species- and virus-specific.

### The CaaX prenylation motif of GBP5 was lost through early stop codon in one lineage of *Vespertilionidae* bats

**Fig 7:**
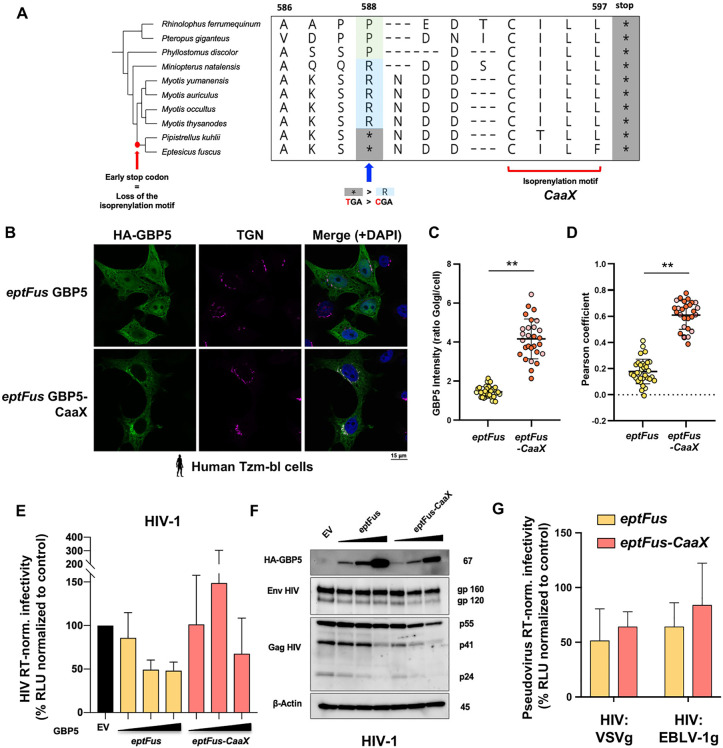
Ancestral reconstruction of the prenylation motif in *Eptesicus fuscus* relocalizes GBP5 to the TGN, but is not sufficient to retrieve anti-pseudoretroviral functions. **A,** Ancestral state sequence reconstruction upstream of the *Eptesicus fuscus-*CaaX prenylation motif. C-terminal end of the protein alignment of the 10 bat GBP5s tested in functional assays (asterisk, stop codon). Phylogenetic tree was used to infer the ancestral sequence of the C-terminal region, the branch where the prenylation motif was lost by a premature stop codon is annotated on the tree. The site of mutagenesis for reconstruction is indicated by the blue arrow. **B,** Reconstruction of the C-ter relocalizes *Eptesicus fuscus* GBP5-CaaX to the *trans-*Golgi network (TGN). Briefly, TZM-bl cells were transfected with plasmids encoding HA-GBP5s and, 48 hour later, were analyzed by confocal fluorescence microscopy. GBP5, nuclei and TGN were stained with anti-HA, DAPI and anti-TGN46 antibodies, respectively. Scale bar indicates 15 μm. **C,** GBP5 mean intensity at the Golgi vs. the total cell was quantified for the wild-type *eptFus* and the mutant *eptFus-CaaX*. Each dot corresponds to one cell. Two independent replicates are identified by different dot colors. **D,** Pearson coefficient correlation per cell calculated between GBP5 and TGN signals for the wild-type *eptFus* and the mutant *eptFus-CaaX.* Data are represented as a mean ± SD. Statistics vs. the corresponding control condition, Nested *t* test: **, *p*-value < 0.01 (*n* = 2). **E–G,** Ancestral reconstruction of the prenylation CaaX did not increase *Eptesicus fuscus* GBP5 restriction of intrinsic viral infectivity. E, Infectivity of RT-normalized HIV-1 pseudotyped-viruses in the presence of GBP5, normalized to the condition without GBP5 (EV control) at 100%. Dose of GBP5 plasmids: 1, 2, and 4 µg with constant total DNA transfected across conditions. Experimental setup as in Fig 5A. RLU, Relative light units. Viral titers (RT activity) are shown in S6 Fig. F, Corresponding western blot showing the expression of HA-GBP5, HIV-1 Env and Gag in the viral producer Tzm-bl cells with beta-actin as loading control (kDa, on the right). Quantification of three independent experiments is shown in S7 Fig. G, Intrinsic infectivity of (RT-normalized) VSVg or EBLV-1g pseudotyped retroviruses in the presence of GBP5s, normalized to EV control at 100%. Experimental setup as in Fig 6A.**, *p*-value < 0.01 (versus control). The data underlying this Figure can be found in S2 Dataset.

To identify molecular determinants of the species-specific subcellular distribution and antiviral activity of bat GBP5s, we performed comparative phenotypic and phylogenetic analyses. Remarkably, we uncovered that several species (*n* = 6) from the same taxon in the *Vespertilionidae* family share an early, fixed stop codon in their genetic sequence (Figs 7A and S2, complete alignment: see Data availability). This includes *Pipistrellus kuhlii* and *Eptesicus fuscus,* which have remarkably different subcellular localization and reduced antiviral functions compared to others (Figs 4–6). Importantly, beyond data from publicly available sequences, we obtained and treated *Eptesicus fuscus* cells with universal type I interferon, extracted total RNA and performed RT-PCR and Sanger sequencing to de novo sequence the IFN-induced GBP5 from mRNA transcripts (see Materials and methods). This confirmed the presence of an early stop codon. The acquisition of the latter certainly occurred through a single nucleotide mutation (C to T) in the common ancestor of this bat clade, approximately 22.6 million years ago (MYA), leading to a nonsynonymous change: “CGA” coding for Arginine (R) to “TGA” Stop codon (Fig 7A). Of note, the nucleotide sequence of the corresponding GBP5 mRNA transcripts, after the early stop codon, did not greatly diverge (Figs 7A and S8, complete alignment available), confirming a relatively recent emergence of this mutation. Interestingly, this natural change led to the complete loss of the prenylation CaaX motif in the GBP5 C-terminal region (Figs 7A, S2, and S8, complete alignment available) and this correlated with different subcellular localization and antiviral functions.

### Ancestral reconstruction of the prenylation motif in *Eptesicus fuscus* relocalizes GBP5 to the TGN in human cells, but does not affect pseudo-retrovirus intrinsic infectivities

The loss of prenylation signal in another unrelated ISG, the OAS1 (2′-5′-oligoadenylate synthetase 1) protein, was recently described in *Rhinolophidae* bats and was associated with a loss of antiviral activity against SARS-CoV-2 [41]. Here, we reconstructed the C-terminal part of GBP5 to rescue its prenylation motif. We used site-directed mutagenesis of *Eptesicus fuscus* GBP5-encoding plasmid to replace the early stop codon with the ancestral amino acid arginine (Fig 7A, *Eptesicus fuscus-*GBP5-CaaX), restoring a long GBP5. Interestingly, while *Eptesicus fuscus* GBP5 had a cytoplasmic nucleus diffused distribution (Fig 4), the repair of the prenylation motif led to its relocalization to the TGN (Fig 7B–7D for quantification of immunofluorescence analyses), thereby restoring the ancestral subcellular localization.

Next, we investigated the impact of *Eptesicus fuscus-*GBP5-CaaX on the infectivity of the diverse pseudoviruses previously tested in this study. Interestingly, the rescue of the CaaX motif was not sufficient to enhance the antiviral effect of *Eptesicus fuscus* GBP5 against the envelope glycoproteins of HIV, EBLV-1, and VSV (Fig 7E–7G). Moreover, *Eptesicus fuscus-*GBP5-CaaX, despite being in the TGN, did not induce retention of immature HIV gp160 and had no effect on gp160:gp120 ratios (Figs 7F and S4–S7). The CaaX-reconstructed GBP5 and wt eptFus GBP5 were similar regarding their effect on HIV-1 Gag and Env total protein expression levels, without impact on cell viability (Figs 7F and S5–S7).

Therefore, the reconstruction of the ancestral CaaX prenylation motif of *Eptesicus fuscus* GBP5 led to subcellular relocalization in human cells. Yet, it was not sufficient to restore the tested antiviral activities on the viral glycoproteins, suggesting that other regions/determinants, beyond CaaX, of *Eptesicus fuscus* GBP5 have evolved and are implicated in this antiviral mechanism.

### Ancestral loss of GBP5 prenylation motif in a lineage of bats led to its subcellular relocalization in *Eptesicus fuscus* bat cells and increased restriction against rhabdovirus

**Fig 8:**
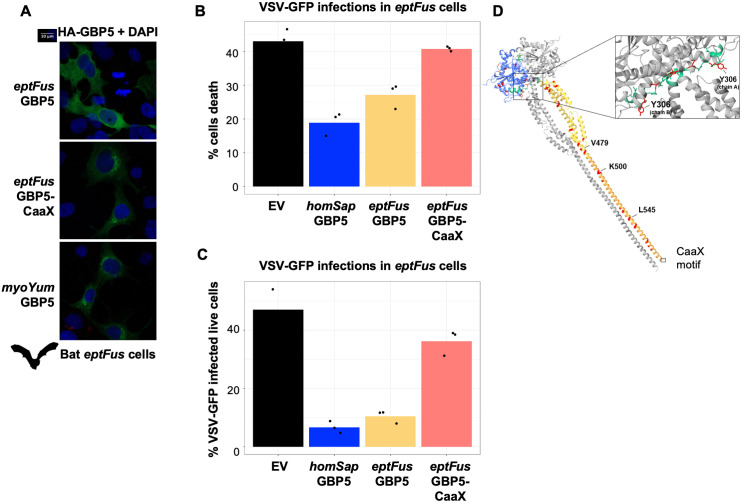
Ancestral reconstruction of the prenylation motif in *eptFus* GBP5 leads to its subcellular relocalization in *Eptesicus fuscus* cells and to a loss of the capacity to restrict VSV-GFP infections. **A,** *Eptesicus fuscus* cells were transfected with plasmids encoding HA-GBP5s and, 48 hours later, were analyzed by confocal fluorescence microscopy with anti-HA antibody. Nuclei were stained with DAPI. Of note, anti-TGN46 antibody did not cross-react in bat cells. MyoYum GBP5 was also transfected as a control of TGN subcellular localization. **B** and **C,** VSV-GFP infections of eptFus bat cells expressing or not GBP5s: total % of cell death (B) and % of VSV-GFP infected live cells as measured by flow-cytometry. Each point corresponds to an independent replicate. **D,** 3D protein structure prediction (AlphaFold) of the reconstructed *Eptesicus fuscus*-*CaaX* GBP5 dimer. Colored and gray chains each correspond to a monomer. Blue, GTPase domain. Green, hinge domain. Yellow, middle domain. Orange, catalytic domain. Red, residues different from *Myotis yumanensis*. Credit: https://www.phylopic.org/. The data underlying this Figure can be found in S2 Dataset.

To test *Eptesicus fuscus* bat GBP5 functions in the context of bat cells, we successfully exogenously expressed myoYum GBP5, eptFus GBP5, and the CaaX-reconstructed mutant in the *Eptesicus fuscus* bat cell lines by DNA transfection (of note, in the absence of IFN stimulation, the endogenous GBP5 mRNA transcript is not detectable in these cells). First, through confocal immunofluorescence imaging, we found that *eptFus* GBP5 expression was diffused in its own species cells and that it re-localized to a specific compartment upon CaaX reconstruction similar to the myoYum GBP5 (of note, the TGN antibody did not cross-react in *Eptesicus* bat cells) (Fig 8A), suggesting a similar subcellular phenotype in these bat cells than in the human ones.

Second, we tested the production of infectious pseudoretroviral particles in these *Eptesicus fuscus*, as well as *Myotis yumanensis*, bat cells in +/− GBP5 conditions. However, the production of infectious virions was intrinsically restricted in these cells, as noted by the absence of Gag maturation (only p55 is expressed) and the detection of Env expression in cell lysates (S9 Fig), as well as absence of detected RT activity in supernatants. Therefore, we finally tested another viral infection in the *Eptesicus fuscus* bat cells, using full-length infectious rhabdovirus VSV-GFP. Briefly, bat cells were seeded and transfected 48 hours later with 2 µg of EV or GBP5 plasmids in 6-well plates. Thirty-two hours later, cells were infected with equal amounts of VSV-GFP. 16 hours post-infection, cells were fixed for flow-cytometry analyses and we quantified cell death (Fig 8B) and GFP+ infected living cells (Fig 8C). We found that human GBP5 protected against VSV-induced cell death and VSV-GFP infections, confirming its antiviral activity against VSV [19] even in bat cells. Moreover, we showed that eptFus GBP5 also had antiviral activity against VSV-GFP. Finally, we found that the eptFus GBP5 with the CaaX reconstruction did not protect against VSV-GFP-induced cell death and did not restrict VSV-GFP infection. This shows that the loss of this motif is necessary for eptFus GBP5 antiviral function against VSV.

Altogether, our study supports an evolutionary model in which the loss of the C-terminal CaaX motif in GBP5 in the common ancestor of *Pipistrellus* and *Eptesicus* bats, ~22 MYA, led to changes in GBP5 subcellular localization and may have been beneficial (i.e., selected) in the context of ancient Rhabdoviral-like infections, thanks to its increased antiviral activity.

## Discussion

In this study, we found that bat GBP5 displays signatures of genomic and functional adaptations conferring specificities to bat antiviral innate immunity. Our integrative approach supports a model in which bat GBP5 has been engaged in a molecular conflict with viral pathogens, including ancient rhabdoviral-like infections, during Chiroptera evolutionary history. Indeed, we found evidence of signatures of positive selection, genomic duplications and natural C-terminal truncation of the defense protein. And importantly, these genomic adaptations contributed to variations in the subcellular localization of bat GBP5 and to species-specific adaptations against modern RNA viruses. Because GBP5 was the most differentially expressed gene upon interferon stimulation of *Myotis yumanensis* primary cells derived from multiple individuals and because we studied bat GBP5 cellular and antiviral functions, notably from *Eptesicus fuscus*, in the corresponding bat species cells, our experimental design strongly supports our primary findings of bat GBP5’s importance in viral infections.

Combining genomic and functional studies, we revealed the natural loss of the prenylation motif in GBP5 of six bat species from the same taxon, which led to the loss of localization to the TGN and increased restriction against a rhabdovirus (VSV-GFP) infection, at least in the context of *Eptesicus fuscus* bat cells. This loss happened in the common ancestor around 22 MYA by missense mutation leading to an early stop codon. The loss of prenylation motif in a protein was previously reported in another important innate immune factor, the dsRNA sensor OAS1, in the bat *Rhinolophidae* family; yet, the genetic mechanism of loss was different because the latter was due to an ancestral LTR insertion [41]. In that case, the loss of OAS1 prenylation induced a loss of sensing and restriction against SARS-CoV-2. Because *Rhinolophidae* bats are reservoir species of sarbecoviruses, OAS1 prenylation loss may have been a specific adaptation allowing tolerance (i.e., a lesser negative inflammatory response) to a large diversity of coronaviruses circulating in these bats. It is also possible that OAS’s anti-coronavirus function was lost as a consequence of lesser pathogenic coronaviruses circulating in these bat species. GBP5 prenylation loss may also be an adaptation in response to viral infections specific to this Vesper bat lineage (comprising *Eptesicus* and *Pipistrellus*), which are reservoirs for a diverse range of viruses, including retroviruses and RNA viruses such as rhabdoviruses [42]. Because the loss of Cter in GBP5 was beneficial in the context of VSV-GFP infection in *Eptesicus fuscus* cells, it suggests possible selection in response to a rhabdoviral-like epidemic about 22 MYA.

The ancestral reconstruction of *Eptesicus* GBP5 C-terminal with the prenylation motif modified its subcellular localization but, in contrast to *Rhinolophidae* OAS1 [41] and expectations, did not change effect on viral glycoproteins in human cells, suggesting that other interfaces of GBP5 may have also diverged. These determinants may be amongst the sites that have evolved under strong positive selection, as a possible result of other viral pressures. For example, sites under positive selection in bat PKR pointed to molecular interfaces, determinants and conflicts with poxvirus antagonists [8]. In the present work, we found clear species-specificity in heterologous virus-host assays (as reviewed in [43]), further supporting that some positively selected sites may be directly involved in antiviral activity. To start looking for the possible determinants, we compared the genetic sequences of GBP5 *Eptesicus fuscus-*CaaX and *Myotis yumanensis*, which share 94.7% amino acid (aa) identity but have different phenotypes (i.e., *Eptesicus fuscus-*CaaX GBP5 has little antiviral function while *Myotis yumanensis* GBP5 has strong antiviral effects) (S2 and S8 Figs). We identified several divergent residues, highlighted in red on the 3D predictive structure of *Eptesicus fuscus*-CaaX (Fig 8D), including three of them, V479, K500, and L545, that have evolved under strong positive selection in bats (Fig 3C, 3D). Genetic mutations appearing at these sites, potentially resulting from virus-host genetic conflicts, could be key interfaces involved in the GBP5 antiviral mechanisms. Furthermore, an additional site Y306, located in the hinge domain, is essential for the conformational change of human GBP5 (in which an aspartic acid is found at the same position), enabling human GBP5 dimerization and its antiviral activity [40]. This mutation could therefore impair correct folding of the protein and compromise its active state, although evidence of anti-VSV activity suggest otherwise.

Another case of such functional-genetic comparative analysis is possible amongst the four closely related *Myotis* species, which have different antiviral phenotypes and only 13 divergent sites between them (S10 Fig). Indeed, *Myotis occultus* showed a stronger restrictive effect against HIV:VSVg than the other *Myotis* species (≃80%, versus 60% of restriction for *Myotis thysanodes*, for example). Residue K547 is the only residue that differentiates *Myotis occultus* from other functionally tested *Myotis* (also different from human GBP5), suggesting a potentially interesting candidate for future functional analysis. Collectively, comparative analysis with *Myotis yumanensis* revealed several residues that would be interesting to functionally test. Further studies are needed to identify all molecular determinants of bat GBP5 antiviral activity. It would also be interesting to investigate potential viral countermeasures, and experiments at different time points and with suspected viral antagonists could help decipher this.

Bat GBP5 restricts virion infectivity in a species-specific manner, probably affecting cleavage and glycosylation of HIV envelope glycoproteins. Human GBP5 activity is linked to cellular furin-dependent inhibition for HIV glycoprotein cleavage, whereas impaired glycosylation is mediated by a furin-independent mechanism [16,18,19]. Recent investigations on SARS-CoV-2 revealed that human GBP5 may affect the activity of the oligosaccharyltransferase (OST) complex, suggesting that any proteins glycosylated by the OST complex in the ER may be impacted by GBP5 [21]. Glycoproteins of HIV, EBLV-1, and VSV could interact with this cellular partner and depend on its activity for their glycosylation. Given the inter-species antiviral specificities, it would be interesting to explore these two mechanisms by furin activity and peptide N-glycosidase (PGNase) assays. Furthermore, although the GTPase activity of human GBP5 appeared dispensable to its described antiviral activities [16], investigating the involvement of bat GBP5 GTPase activity in antiviral activity could reveal new features. Because we observed a slight repression of HIV Gag p41 and p24 structural protein expressions in a CaaX motif-independent manner, it is possible that the enzymatic activity of GBP5 may be required for complementary antiviral activities. Furthermore, some bat GBP5s seem to restrict more viruses than human GBP5. It would therefore be interesting to test other viruses and to determine whether bat GBP5s have acquired an extended antiviral breadth and by which mechanism. Given bat diversification and *GBP* genes’ duplications, GBP5 from bats may also have evolved a yet unknown restriction mechanism. Exploring functional impact of natural variations throughout mammalian evolution also contributes to the broad mechanistic understanding of innate immunity.

Beyond the antiviral effector functions, GBP5 also targets a broad spectrum of bacteria and parasites by promoting their eradication via activation of the inflammasome, notably leading to pyroptosis of the infected cell [14,15,44]. Variations at the positively selected sites could also affect these GBP5 functions. Furthermore, rapidly evolving residues in bat innate immune factors have also been involved in increased antiviral defense, or in beneficial viral tolerance. For example, bat IRF3 positive selection at specific serine residue participated in enhancing antiviral defenses [45], or rapid evolution of S358 in bat STING participated in increasing viral tolerance [46], respectively. Previous studies also reported that altered expression and function of bat inflammasome components, leading to reduced inflammasome activation, may participate to traits associated with asymptomatic viral reservoirs and greater longevity [3,47]. Some bat GBP5 adaptations could therefore also be implicated in dampened inflammatory responses, providing interesting insights for understanding viral tolerance mechanisms.

Limitations of the study: previous studies on the IFN stimulation of bat cell lines [22–24], including from *Myotis* bats, also used universal type I IFN; yet it would be interesting to test the corresponding bat species interferons. Furthermore, it would be interesting to test the effect of knock-out of endogenous IFN-induced GBP5 in the bat cells in the context of viral infections. However, several challenges remain, such as CRISPR setup in relevant bat cells, efficient viral infections, including of VSV in the context of IFN treated bat cells (as GBP5 is only expressed in IFN condition).

Overall, this study contributes to a better understanding of bat innate immunity, which has been a prime objective. Past pathogenic viral exposures at different times led to modern antiviral immune specificities. This study also demonstrates the importance of including multiple related species and viruses in comparative functional studies to better assess divergence in their immune systems, thereby participating to fundamental knowledge of mammalian innate immunity and ultimately contributing to translational research for One Health and therapy [48].

## Materials and methods

### Ethics statement, bat sampling, and generation of cell lines

*Myotis spp. (yumanensis, occultus, auriculus*, and *thysanodes)* bats sampled for this study were wild caught under scientific collection permits from California (California Department of Fish and Wildlife) and Arizona (Arizona Game and Fish Department): AZ SP407155, AZ SP407113, AZ SP403977, SCP-2672, and UA IACUC 20-615. Protocols were validated by Animal Care and Use Committees at UC Berkeley and University of Arizona: IACUC AUP-2020-11-13795, AUP-2023-04-16265, and UA IACUC 20-615. Bats were sampled using standard mist-netting procedures, including taking standard body measurements, following USGS recommendations for White-Nose Syndrome and COVID-19 prevention. Two 3-mm wing punch biopsies were taken from the left and right plagiopatagium of each donor individual and placed in a live cell collection media consisting of DMEM/F12 (Gibco) supplemented with 15mM HEPES (Gibco), 20% FBS (Gibco), and 0.2% puromycin (Invivogen) [49–51]. Wing punches were then brought back to a cell culture facility in Berkeley, where they were used to generate cell lines as previously described [49–51].

### Cell culture

Human embryonic kidney 293T (ATCC, cat. CRL-3216), HeLaP4P5 (HeLa cells expressing CD4^hi^CCR5^hi^CXCR4^hi^), and TZM-bl (NIH AIDS Research and Reference Reagent Program, Cat. 8129; HeLa cells expressing CD4^hi^CCR5^hi^CXCR4^hi^ and the luciferase reporter under an LTR promoter) cells were grown in DMEM supplemented with 5% fetal bovine serum (FCS, Sigma cat. F7524) and 100 U/ml of penicillin/streptomycin (Sigma–Aldrich). Bat *Myotis yumanensis* primary cells were maintained in DMEM supplemented with 10% fetal bovine serum (FCS, Sigma cat. F7524) and 100 U/ml of penicillin/streptomycin (Sigma–Aldrich). Bat *Eptesicus fuscus* immortalized skin fibroblasts (a gift from Cédric Feschotte and Rachel Cosby [52]) were maintained in DMEM supplemented with 7.5% fetal bovine serum and 100 U/ml of penicillin/streptomycin.

### RNA extraction for next-generation sequencing and quality assessment

*Myotis yumanensis* primary cells from the three individuals were plated in 12-well plates. Cells from each individual constituted a replicate. Twenty-four hours later, cells were either treated with universal type I interferon treatment (PBL assay science) at 1,000 U/mL or the medium was replaced with IFN-free DMEM for the mock condition. After 6 hours, cells were collected and pelleted, and then stored at −20 °C. RNA was extracted from frozen cell pellets according to RNA isolation Macherey–Nagel kit protocol. To maximize RNA extraction quality, DNAse incubation time was increased to 30 min. Total RNA concentration was quantified using Qubit RNA high sensibility kit and Qubit4 machine (Thermofisher). RNA integrity was assessed by capillary electrophoresis on Tapestation 4,150 (Agilent). All samples reached an RNA integrity number superior to 9 and were used for library preparation.

### Library preparation and RNAseq

Samples were enriched in mRNA using NEBNext Poly(A) mRNA Magnetic Isolation Module (New England Biolabs, E7490- lot n°10151189) following the manufacturer’s protocol. Enriched mRNA samples were stored at −80 °C prior to library concentration. RNA libraries were generated using CORALL Total RNA-Seq Library Prep kit (UDI 12 nt set B1, Lexogen). Libraries’ control assessments were performed using Qubit dsDNA HS Assay Kit on Qubit4 and capillary electrophoresis Tapestation 4,150. Libraries were then mixed in equimolar concentration. The sequencing was performed using three successive runs on Illumina NextSeq 500 platform in paired-end 78-pb with dual indexing at IGFL sequencing platform.

### Bioinformatic analyses of RNAseq data

#### Data quality assessment. 

Raw data quality was assessed using FASTQC (version 0.12.0) and results were compiled using MultiQC (version v1.14). The data were trimmed to remove adapters/UMI index and low-quality reads using FASTP (version 0.23.1, with the parameter -min_len 40 and -q 20).

#### Mapping and quantification.

Transcriptome mapping was performed using a transcriptome index (Salmon 1.10.2), based on *M. yumanensis* reference transcriptome. As the quasi-mapping transcriptome rate ranged between 40%–55% using Salmon, the reads were also mapped on *M. yumanensis* reference genome using STAR (2.7.11b) to check for potential contamination. The genomic mapping rate had a range between 70%–90%. A manual inspection showed the mapping rate difference was solely due to the unannotated UTR regions in the transcriptome of these nonmodel species. Importantly, it did not affect gene-level differential expression (DE) analyses, because in STAR, reads mapping to unannotated UTR regions do not contribute to gene counts.

#### Table counts and DESeq2 analysis.

Salmon quantification files were imported using tximport (version 1. 26.1) with the option “lengthScaledTPM” and “countFromAboundance”. The gene orthology relationship with *Homo sapiens* was obtained using Orthofinder (version 2.3.10) with four others *Myotis* species and was restricted to genes defined as one-to-one orthologs with *Homo sapiens*. Differentially expressed genes were obtained using DESeq2 (v 1.42.1) package. The following additional package were used to generate the different figures: ggrepel (version 0.9.5), ggplot2 (version 3.5.1), tidyverse (version 2.0.0), and MetBrewer (version 0.2.0).

#### Comparative analyses with Shaw and colleagues 2017 dataset.

Our GBP5 RNAseq results were compared with processed results from Shaw *and colleagues* 2017 (EBI, project accession number PRJEB21332) and available at https://isg.data.cvr.ac.uk/. The cells used for the Shaw *and colleagues* dataset were also primary fibroblast cell lines (i.e., cows, 4 individuals; sheep, 3 individuals; dog, 1 individual; human and rodent, PromoCell and European Collection of Authenticated Cell Cultures: C-12302 and 06090769, respectively; *M. lucifugus*, from Oregon, USA) [22]. For the IFN treatment, cell lines were similarly treated with 1,000 U/ml universal type I IFN for 6 hour, except for dog cell lines treated with 200 ng/ml canine IFNα.

### De novo sequencing of GBP5 genes

Total genomic RNA was extracted from bat primary fibroblast cells of *Myotis auriculus, Myotis occultus, Myotis thysanodes,* and *Myotis yumanensis*, as well as from the *Eptesicus fuscus* immortalized skin fibroblasts, using Macherey–Nagel NucleoSpin RNA kit following the manufacturer’s protocol. Total RNA was reverse transcribed into complementary DNA (cDNA) with random primers and oligo(dT), using the SuperScript III One-Step reverse transcription polymerase chain reaction (PCR) kit (Thermo Fisher Scientific, Poland). GBP5 mRNA was then amplified from each species using 10 ng of cDNA and different sets of primers (S2 Table) that were specifically designed using an alignment of publicly available GBP5 sequences. The PCR reactions were performed using the New England Biolabs (NEB) Q5 High-Fidelity DNA Polymerase, following the manufacturer’s protocol, including a final volume of 50 µl, a 0.5 µM primer concentration, and an annealing temperature of 61 °C. PCR products with multiple bands were excised and purified from gel using the NucleoSpin Gel and PCR Clean-up Kit from Macherey–Nagel. Sanger sequencing of GBP5 was performed by a commercial company (Genewiz, Azenta Life Sciences, Germany).

### Collection of GBP5 orthologous sequences

Homologs of all six human GBPs were searched in 536 mammalian reference genomes (downloaded from NCBI in June 2023) by sequence similarity using the miniprot software [53]. Homologous matches that were at least 50% of the length of the human GBP5 were selected. Because genome sequences can include indel errors that destroy the reading frame, a first round of multiple sequence alignment of the retrieved GBP homologs was run with MACSE v2 [54]. MACSE uses a statistical model that takes frameshifts into account in order to properly align homologous codons. The resulting coding sequences were then processed with PREQUAL [55] to eliminate segments with insufficient homology and likely to be misaligned. Then, the remaining coding sequences were aligned with MACSE again. The GBP5 orthologs to the human GBP5 were then identified by generating a phylogenetic tree with IQ-TREE2 (under a GTR + R6 model as identified using ModelFinder) [29].

In total, GBP5 coding sequences from Chiroptera (*n* = 55; including 5 newly generated sequences), Primates (*n* = 37), Rodentia (*n* = 80), Artiodactyla (*n* = 97), Carnivora (*n* = 51), Lagomorpha (*n* = 10), Perissodactyla (*n* = 9), Proboscidea (*n* = 3), Pilosa (*n* = 1), Cingulata (*n* = 4), and Xenarthra (*n* = 1) were retrieved.

### Phylogenetic and positive selection analyses of GBP5 orthologous sequences

GBP5 orthologous codon sequences were aligned for each mammalian group separately using PRANK [28] or Muscle [56], and were manually curated. A phylogenetic tree was built using the maximum likelihood method implemented in IQ-TREE using ModelFinder to identify the best substitution model [29]. Node statistical support was computed through 1,000 bootstrap replicates. The gene codon alignments were then submitted to positive selection analyses using the HYPHY package (either through command line or the DataMonkey webserver [35,39]). First, Branch-site Unrestricted Statistical Test for Episodic Diversification (BUSTED, [30]) was used to determine whether the gene has experienced episodic positive selection (*p* value < 0.05). Second, an adaptive branch-site REL test for episodic diversification (aBSREL) [34] was used to identify the branches under positive selection with a cutoff at *p* value < 0.1 as well as to quantify the dN/dS ratio for each branch independently (the tree was newly generated in aBSREL). Lastly, we used three statistical analyses to identify the specific sites under positive selection: MEME, which detects individual sites subject to episodic diversifying selection ([37], cutoff *p* value < 0.01), FUBAR (A Fast, Unconstrained Bayesian AppRoximation for Inferring Selection, [36]; cut off posterior probabilities PP > 0.90) and FEL (Fixed Effects Likelihood, [38]; cutoff *p* value < 0.05).

### Plasmids

GBP5s were amplified from bat cDNA (species: *Myotis auriculus, Myotis occultus, Myotis thysanodes, Myotis yumanensis*, and *Eptesicus fuscus)* of fibroblast cells (details above) or synthesized by a commercial company (Genewiz, Azenta Life Sciences, Germany) (species: *Rhinolophus ferrumequinum, Pteropus giganteus, Pipistrellus kuhlii, Miniopterus natalensis*, and *Phyllostomus discolor*). They were cloned (*via* BstBI and XhoI restriction sites) into the vector MT06 (RRL.sin.cPPT.CMV/-BamHI-HA-BstBI-E2-crimson-XhoI.IRES-puro.WPRE, shared by Caroline Goujon: Addgene plasmid # 139448 [57]), where an HA tag was added between BamHI and XhoI restriction sites. Ancestral reconstruction of the *Eptesicus fuscus* isoprenylation motif expression was generated from wild-type *Eptesicus fuscus* pMT06-GBP5 by replacing the Stop codon with corresponding ancestral residue, using the Quik Change Lightning Site-Directed Mutagenesis Kit (Agilent) following the manufacturer’s instructions (set primers available in S2 Table). For pseudovirus production, a plasmid coding for LAI-∆Env-Luc2 genome (Bru-∆Env-Luc2 that has no Env and encodes a firefly luciferase in place of the nef gene, shared by Michael Emerman) was used and pseudotyped with HIV-1 NL4.3 Env (along an HIV-1 Rev plasmid to facilitate nuclear export of Env, [58]), VSVg (pMD2.G, Didier Trono: Addgene plasmid # 12259) or European bat lyssavirus EBLV-1 Env (pLTR-EBLV1env, a gift from Daniel Sauter [18]).

### HIV-1 production and infection assay

A total of 4 × 10^5^ HEK-293T cells were seeded in 6-well plates and transfected the next day with plasmids coding for LAI-∆Env:Luc2 genome (1.2 µg), NL4.3 envelope (120 ng), HIV-1 Rev (75 ng), as well as indicated doses of HA-GBP5 (0, 1, 2, and 4 µg) or empty vector EV control (expressing E2-crimson) using HBS/CaCl2 to produce infectious HIV-1 single-round viruses. Forty-eight hours later, cells were harvested for western blot and virus-containing supernatants were clarified for virus titration, by measuring HIV-1 RT activity (see below), western blot (see below) and infections. For infection, 1 × 10^4^ HeLaP4P5 were seeded into white-clear bottom 96-well plates. Twenty-four hours later, cells were infected with 30 mU RT of virus supernatant in the presence of DEAE-Dextran solution (20 µg/ml). Luciferase activity from the reporter HIV-1: Luc2 virus was measured 72 hours post-infection by Tecan Spark Luminometer.

### VSV and EBLV-1 pseudovirus productions and infection assays

A total of 4 × 10^5^ HEK-293T cells were seeded in 6-well plates and transfected the next day with Bru-∆Env-Luc2 (shared by Michael Emerman) (1.2 µg), VSV-g or EBLV-1 envelope, as well as indicated doses of HA-GBP5 or empty vector control using HBS/CaCl2 to produce infectious pseudotyped virus. Virus-containing supernatant was harvested, clarified for viral titration, by measuring RT activity and infection. For infection, 1 × 10^4^ HEK-293T were seeded into white-clear bottom 96-well plates and infected with 50 mU RT of virus supernatant. Luciferase activity from the reporter pseudovirus was measured 72 hours post-infection by Tecan Spark Luminometer.

### Virus titration by RT activity

Titration of HIV-1, VSVg and EBLV-1 Env pseudoviruses from supernatant were quantified by measuring reverse transcriptase (RT) activity as in [59]. Briefly, virions were lysed in a homemade lysis buffer (0.25% Triton X-100, 50 mM KCl, 100 mM Tris−HCl pH 7.4, 40% glycerol) and 0.4 U/μL RiboLock RNase inhibitor (Thermo Scientific). The viral lysates were then diluted in H2O and added to the RT-qPCR mix: 35 nM bacteriophage MS2 RNA (Roche) as a template for RT, 100 nM of each primer (5′-TCCTGCTCAACTTCCTGTCGAG-3′ and 5′-CACAGGTCAAACCTCCTAGGAATG-3′) and Takyon No ROX SYBR 2X MasterMix blue dTTP buffer (Eurogentec) in a total reaction volume of 20 μL. Purified RT enzyme (Abnova) was used to make the standard curve. The RT–qPCR reaction was carried out in a Bio-Rad CFX96 cycler with the following parameters: 42 °C for 20 min, 95 °C for 5 min and 40 cycles (95 °C for 5 s, 60 °C for 30 s and 72 °C for 15 s).

### Viability measurement

A total of 1 × 10^4^ HEK-293T cells were seeded in white-clear bottom 96-well plates and transfected the following day with plasmids encoding the LAI-∆Env:Luc2 genome (30 ng), NL4.3 envelope (3 ng), HIV-1 Rev (1.9 ng), as well as a plasmid encoding HA-GBP5 (100 ng) or an empty vector control using TransIT-LT1 (Mirus). Of note, the quantities were calculated to maintain the same ratio used for HIV production with the higher dose (4 µg) of GBP5 described above. Forty-eight hours later, cell viability was assessed by measuring adenosine triphosphate (ATP) levels using the CellTiter-Glo Luminescent Cell Viability Assay (Promega). Treatment with etoposide (100 µM) for 24 hour was used as a positive control (Sigma). The luminescence of ATP activity was quantified using a Tecan Spark luminometer.

### Western blot and antibodies

Cells were lysed by ice-cold RIPA buffer (50 mM Tris pH8, 150 mM NaCl, 2 mM EDTA, 0.5% NP40) supplemented with protease inhibitor cocktail (Roche) and by sonication. Cell-free virions were concentrated and purified from the supernatant by centrifugation (2 hour, 30,000 rpm, 4 °C) through a 20% sucrose cushion and resuspended for lysis in Exo-RT lysis buffer. Proteins from cell or virus lysates were mixed in Laemmli buffer, heated at 95 °C for 5 min and separated by SDS-PAGE on 4%–20% Bis-Tris gels (Invitrogen) before being transferred to PVDF membrane by an overnight wet transfer at 4 °C. Membranes were blocked in a TBS-T 1X solution (« Tris Buffer Saline », Tris HCl 50 mM pH8, NaCl 30 mM, 0.05% of Tween 20) with 5% powder milk. Proteins were stained with a mouse anti-p24 HIV-1 CA (1:1000, NIH HIV Reagent Program, cat.183-H12-5C), a mouse anti-HIV-1 Env (16H3) (1:1000, NIH-ARP, cat. 12559), a rabbit anti-HA (1:5000, Sigma, cat. H6908), a mouse anti-tubulin (1:5000, Sigma, cat. T5168) or a mouse anti-β-actin (1:5000, Sigma, cat. A2228), and then with a donkey anti-rabbit-HRP (1:5000, Sigma, cat.AP188P), or a donkey anti-mouse-HRP secondary antibody (1:5000, Sigma, cat.AP16017). Detection was made with SuperSignal West Pico Chemiluminescent Substrate (ThermoFisher Scientific) using the Chemidoc Imagina System (Biorad).

### Immunofluorescence imaging of human and eptFus bat cells

A total of 3 × 10^4^ TZM-bl cells or 2.5 × 10^4^ eptFus bat cells were seeded on glass coverslips and were transfected with 1 µg of GBP5 plasmid with jetPRIME (Polyplus) or Lipofectamine 3,000, respectively, according to the manufacturers’ instructions. Forty-eight hours later, cells were fixed with 4% paraformaldehyde (PFA) (Sigma) for 15 min at room temperature and permeabilized in 0.25% Triton-TX100 (Sigma) for 5 min. A blocking step was carried out for 1 hour at room temperature with 3% BSA (Sigma) and 0.1% Triton TX100 in PBS. Primary antibody incubation was carried out for 1 hour at RT with rabbit anti-HA (1:500, Sigma, cat. H6908) and sheep anti-TGN46 (1:500, Biorad, cat.AHP500GT; not cross-reacting in bat cells) to label respectively HA-GBP5 proteins and the TGN. Primary antibodies were detected with secondary donkey anti-rabbit AlexaFluor-594 (Invitrogen, cat.A212027) and donkey anti-sheep AlexaFluor-647 (Thermofisher, cat.A-21448), for 1 hour at RT. All cells were labeled with DAPI (4′,6-diamidino-2-phenylindole) (Thermo Scientific) - containing solution (1:10,000 dilution in PBS). Images were acquired using Zeiss LSM 980 AiryScan (human cells) or LSM 800 (bat cells) confocal microscopes and analyzed with Imaris software (human cells). For quantification analyses, segmentation of the cells was first performed using Cellpose algorithm (version 3.0.11) [60]. Proportion of GBP5 at the TGN and colocalization analysis were then performed on ImageJ software using an automatized macro or the BIOP JACoP plugin, respectively.

### Infection by VSV-GFP in bat cells exogenously expressing GBP5s

Bat *Eptesicus fuscus* immortalized cells were all seeded at 5 × 10^4^ cells/ml in 6-well plates (final volume of 2 ml) at day 1. 48 hours later, they were transfected by lipofectamine 3,000 in three independent experiments with 2 µg DNA of EV or GBP5 plasmids per condition. Thirty-two hours later, cells were infected with equal amounts (6 µl) of VSV-GFP [61], whose stock was made from 293T cells. 16 hours post-infection, eptFus cells were fixed with Fix and Perm Cell Permeabilization kit (Invitrogen, Thermo Fisher Scientific) for flow-cytometry analyses using MacsQuant cytometer (SFR BioSciences) and we quantified cell death and GFP+ infected living cells.

### Other software and statistical analyses

Differences between conditions were statistically analyzed using GraphPad Prism 9 software or R. A Nested *t* test was used for immunofluorescence analysis (Fig 8). One-way ANOVA with Dunnett’s test for all other datasets. For each of these tests, the *p*-value was considered significant when inferior to 0.05: *, *p* value < 0.05, **, *p* value < 0.01, ***, *p* value < 0,001. Error bars in graphics are SD from three independent experiments.

## Supporting information

S1 Fig Nucleotide sequence divergence of bat GBP5s. Bat GBP5 sequences were aligned with MUSCLE and percentage of identity measured with GeneiousR10.
(PDF)

S2 Fig Alignment of functionally tested bat GBP5s. Alignment of functionally tested amino acid sequences from the bat GBP5. In red, positively selected sites in Fig 3C.
(PDF)

S3 Fig Natural variation in subcellular localization of bat GBP5. TZM-bl cells were transfected with a plasmid coding for indicated HA-GBP5 species proteins. Two days post-transfection, GBP5 localization was analyzed by confocal fluorescence microscopy. Nuclei and *trans-*Golgi-network (TGN) were stained with DAPI and anti-TGN46, respectively. Scale bar indicates 15 μm.
(PDF)

S4 Fig Species-specific restriction of bat GBP5 on HIV-1 Env glycoprotein maturation and viral protein expression. **A,** HIV-1 titers in the supernatants as quantified by RT activity (mU/ml) in the context of a dose of HA-GBP5 (1, 2, or 4 µg) or control vector (EV). The corresponding species of GBP5 is shown (name follows the UCSC nomenclature, three letters from genus followed by three letters from species). From three independent experiments, bars are SD. **B,** western blot analysis of HA-GBP5, HIV-1 Env, and HIV-1 Gag, and beta-actin (loading control) from the lysates of the HIV-1 producer cells (bottom) and the purified virion fraction of the supernatant (top) in the context of 4 µg of the corresponding GBP5 or control vector (EV). The data underlying this Supplementary Figure can be found in S2 Dataset.
(PDF)

S5 Fig Expression of bat GBP5 in the context of pseudoviral particle production does not increase cytotoxicity in HEK-293T cells. **A,** Western.blot quantification of three independent experiments of Tubulin, as in Fig 5D. **B,** HEK-293T cells were transfected with plasmids coding for HA-GBP5 or the empty vector (EV control), and for HIV-1 LAI genome and Luciferase reporter (Bru∂EnvLuc2 vector), NL4.3 Envelope and Rev (identical conditions used for HIV infection in Fig 5). 48 hours post-transfection, cell viability was determined by measuring the level of adenosine triphosphate (ATP). A treatment with etoposide (100 µM) during 24 hour was used as a positive control. Mean values of three independent experiments are shown. Statistics versus the corresponding control condition: *, *p* value < 0.05). The data underlying this Supplementary Figure can be found in S1 Dataset.
(PDF)

S6 Fig Titration of pseudoviruses by RT activity in the supernatant. Titration of lentiviruses pseudotyped with EBLV-1g **(A)**, VSVg **(B)**, or HIV-1 Env **(C)** from supernatants, quantified by RT activity (mU/ml). A and B, with 2 µg of HA-GBP5 or control vector (EV) for the corresponding indicated species (i.e., HomSap, Homo sapiens). C, in the context of a dose of HA-GBP5 (1, 2, or 4 µg) or control vector (EV). EptFus-CaaX corresponds to the C-ter ancestral reconstructed *Eptesicus fuscus* GBP5 bearing the CaaX prenylation motif. Left, Normalized values to control (EV) at 1. Right, Raw data (mU/mL). The data underlying this Supplementary Figure can be found in S1 Dataset.
(PDF)

S7 Fig Quantification of western blot from independent experiments, as in Fig 5D, testing the effect of eptFus and the CaaX mutant on viral protein expressions from Fig 7. The data underlying this Supplementary Figure can be found in S2 Dataset.
(PDF)

S8 Fig Alignment of *Myotis yumanensis, Eptesicus fuscus*, and *Homo sapiens* GBP5 protein sequences. (PDF)

S9 Fig Absence of HIV Gag maturation and Env detection in *Myotis yumanensis* bat cell lysates in the context of HIV +/− GBP5. (PDF)

S10 Fig Divergent residues identified between functionally tested *Myotis* GBP5s. Of note, GBP5 from *Myotis occultus* was a stronger restrictor of the infectivity of viral particles bearing VSVg, as compared to other *Myotis* tested (Fig 6).
(PDF)

S1 Raw Images Raw immunoblots of the Figures. **A,** Associated with Fig 5; **B,** Associated with S4 Fig; **C,** Associated with Fig 7; **D,** Associated with S9 Fig.
(PDF)

S1 Table Bat GBP5 species-specific and dose-dependent effects on HIV-1 intrinsic infectivity (upon RT normalization) and RT activity in the supernatant. Summary of the effects of bat GBP5s on the HIV-1 intrinsic infectivity step and on RT activity in the supernatant (“viral production” in the table) relative to the EV control normalized at 100%.
(PDF)

S2 Table Primer sequences to amplify and sequence the GBP5 cDNA from bat cells (“PCR”) and to perform mutagenesis (“Mutagenesis”). (PDF)

S1 Dataset Table of TPM counts from the *Myotis yumanensis* RNAseq from three individuals (Yum2, Yum3, and Yum4) under control and IFN treatment. (CSV)

S2 Dataset Data available from Figs 1E, 1F, 2B, 5B, 5D, 6B, 7C, 7E, 7G, 8B, 8C, S4A, S5A, S5B, S6A–S6C, and S7. (XLSX)

## Abbreviations

aBSREL: adaptive Branch-Site Random Effects Likelihood
ATP: adenosine triphosphate
cDNA: complementary DNA
DE: differentially expressed
EBLV-1: European Bat LyssaVirus 1
FC: fold change
FEL: Fixed Effects Likelihood
GBP: Guanylate-binding protein
GBP5: guanylate-binding protein 5
HIV-1: Human immunodeficiency virus 1
IFITM3: interferon-induced transmembrane protein 3
IFN: interferon
ISG: interferon-stimulated gene
NEB: New England Biolabs
OST: oligosaccharyltransferase
PCR: polymerase chain reaction
PFA: paraformaldehyde
PGNase: peptide N-glycosidase
PKR: protein kinase R
RNAseq: RNA for sequencing
RT: reverse transcriptase
SARS-CoV-2: severe acute respiratory syndrome coronavirus 2
TGN: *trans*-Golgi network
VSV: vesicular stomatitis virus.
