# Correction: Genomic and functional adaptations in the guanylate-binding protein GBP5 highlight specificities of bat antiviral innate immunity

**DOI:** 10.1371/journal.pbio.3003963

**Published:** 2026-08-18

**Authors:** Amandine Le Corf, Sarah Maesen, Amandine Chantharath, Clara Loyer, Juan Manuel Vazquez, M. Elise Lauterbur, Veronika Krchlikova, Lucas Sareoua, Genavieve Gray-Sandoval, Andrea Cimarelli, Carine Rey, Peter H. Sudmant, David Enard, Lucie Etienne

The Data Availability statement for this article is incorrect. The correct Data Availability statement is as follows: All data are publicly available. The scripts are available in GitLab https://gitbio.ens-lyon.fr/ciri/lp2l/gbp5_paper_2025 and in Zenodo https://zenodo.org/records/19208102. The RNAseq data were deposited in the European Nucleotide Archive (ENA) under accession number PRJNA1128057 and the TPM count table is available in Dataset S1. The five de novo GBP5 sequences obtained for five bat species were deposited in GenBank (Accession numbers: PV055693-7). The alignments for phylogenetic analyses are all openly available in FigShare Dataset (https://doi.org/10.6084/m9.figshare.26180785.v1, https://doi.org/10.6084/m9.figshare.26180764.v1, https://doi.org/10.6084/m9.figshare.26180803.v1, https://doi.org/10.6084/m9.figshare.26180761.v1, https://doi.org/10.6084/m9.figshare.26180767.v1, https://doi.org/10.6084/m9.figshare.26180776.v1, https://doi.org/10.6084/m9.figshare.26180794.v1, https://doi.org/10.6084/m9.figshare.26180797.v1), as well as the bat phylogenetic tree from Fig 3A (https://doi.org/10.6084/m9.figshare.30924512) and all results from positive selection analyses (https://doi.org/10.6084/m9.figshare.30924551). Reagents are accessible upon request to partenariats.lyon@inserm.fr.
