# Supplementary figures and images for: Genomic and functional adaptations in the guanylate-binding protein GBP5 highlight specificities of bat antiviral innate immunity
Source: PLoS Biol. 2026 Apr 21;24(4):e3003760. doi: 10.1371/journal.pbio.3003760 (PMC13128109; doi:10.1371/journal.pbio.3003760)

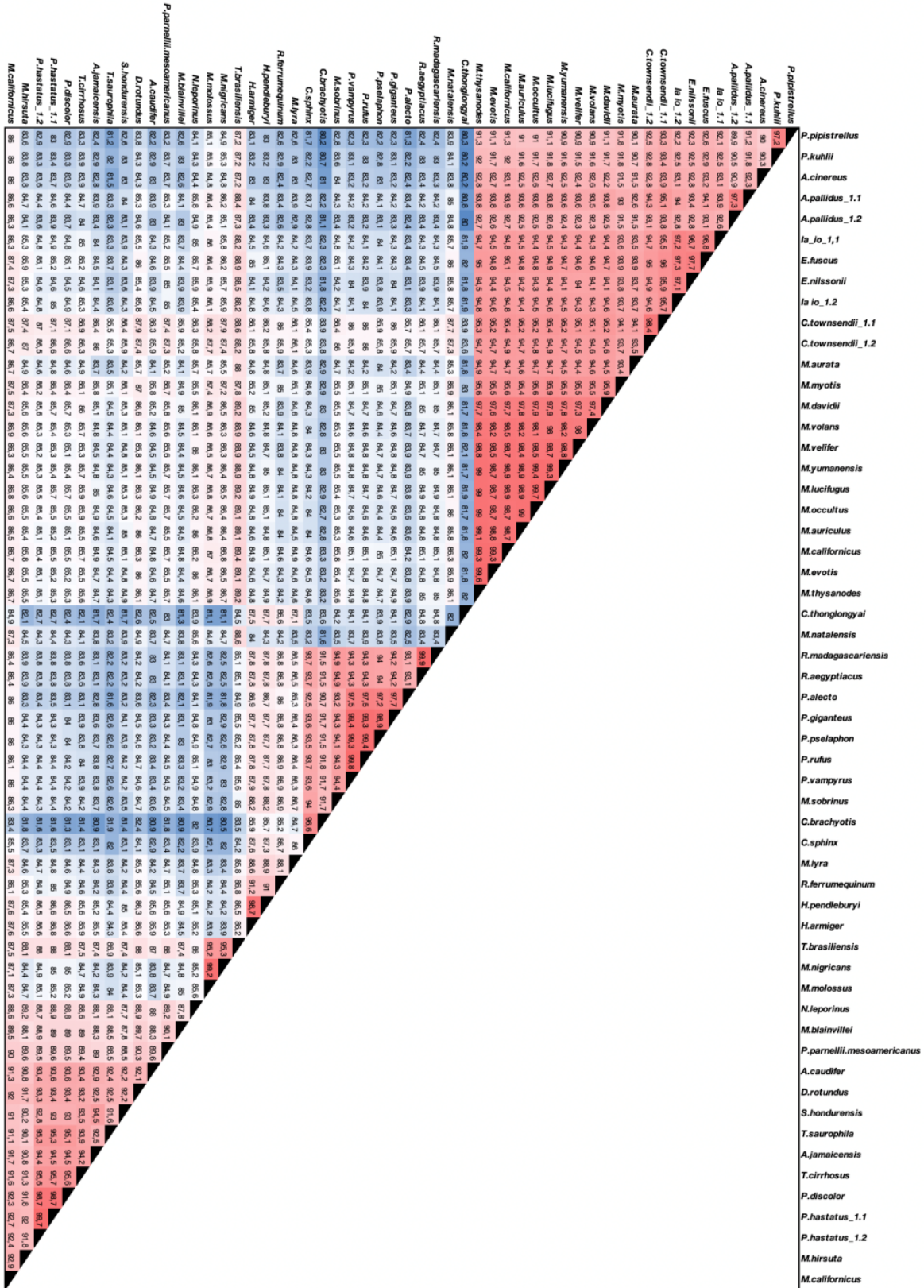

Supplement: S1 Fig — Bat GBP5 sequences were aligned with MUSCLE and percentage of identity measured with GeneiousR10. (PDF) [file pbio.3003760.s001.pdf]
