# Supplementary material for: Genomic and functional adaptations in the guanylate-binding protein GBP5 highlight specificities of bat antiviral innate immunity
Source: PLoS Biol. 2026 Apr 21;24(4):e3003760. doi: 10.1371/journal.pbio.3003760 (PMC13128109; doi:10.1371/journal.pbio.3003760)

Figure S2. Alignment of functionally tested bat GBP5s. Alignment of functionally tested amino acid sequences from the bat GBP5. In red, positively selected sites in Figure 3C.

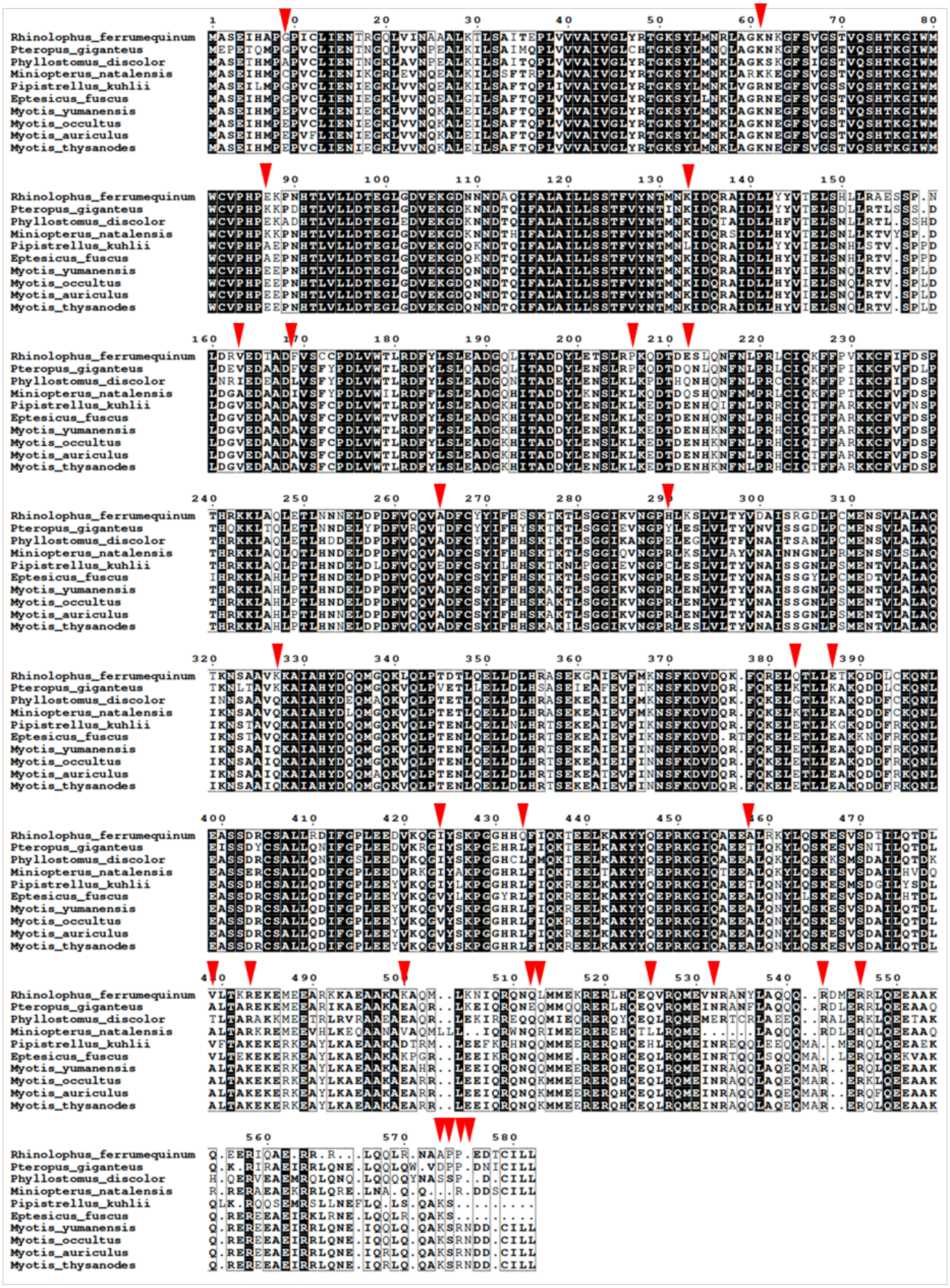

Supplement: S2 Fig — Alignment of functionally tested amino acid sequences from the bat GBP5. In red, positively selected sites in Fig 3C. (PDF) [file pbio.3003760.s002.pdf]
