# Supplementary material for: Genomic and functional adaptations in the guanylate-binding protein GBP5 highlight specificities of bat antiviral innate immunity
Source: PLoS Biol. 2026 Apr 21;24(4):e3003760. doi: 10.1371/journal.pbio.3003760 (PMC13128109; doi:10.1371/journal.pbio.3003760)
Supplement: S3 Fig — TZM-bl cells were transfected with a plasmid coding for indicated HA-GBP5 species proteins. Two days post-transfection, GBP5 localization was analyzed by confocal fluorescence microscopy. Nuclei and trans-Golgi-network (TGN) were stained with DAPI and anti-TGN46, respectively. Scale bar indicates 15 μm. (PDF) [file pbio.3003760.s003.pdf]

Figure S3. Natural variation in subcellular localization of bat GBP5. TZM-bl cells were transfected with a plasmid coding for indicated HA-GBP5 species proteins. Two days post-transfection, GBP5 localization was analyzed by confocal fluorescence microscopy. Nuclei and trans-golgi-network (TGN) were stained with DAPI and anti-TGN46, respectively. Scale bar indicates 15 µm.

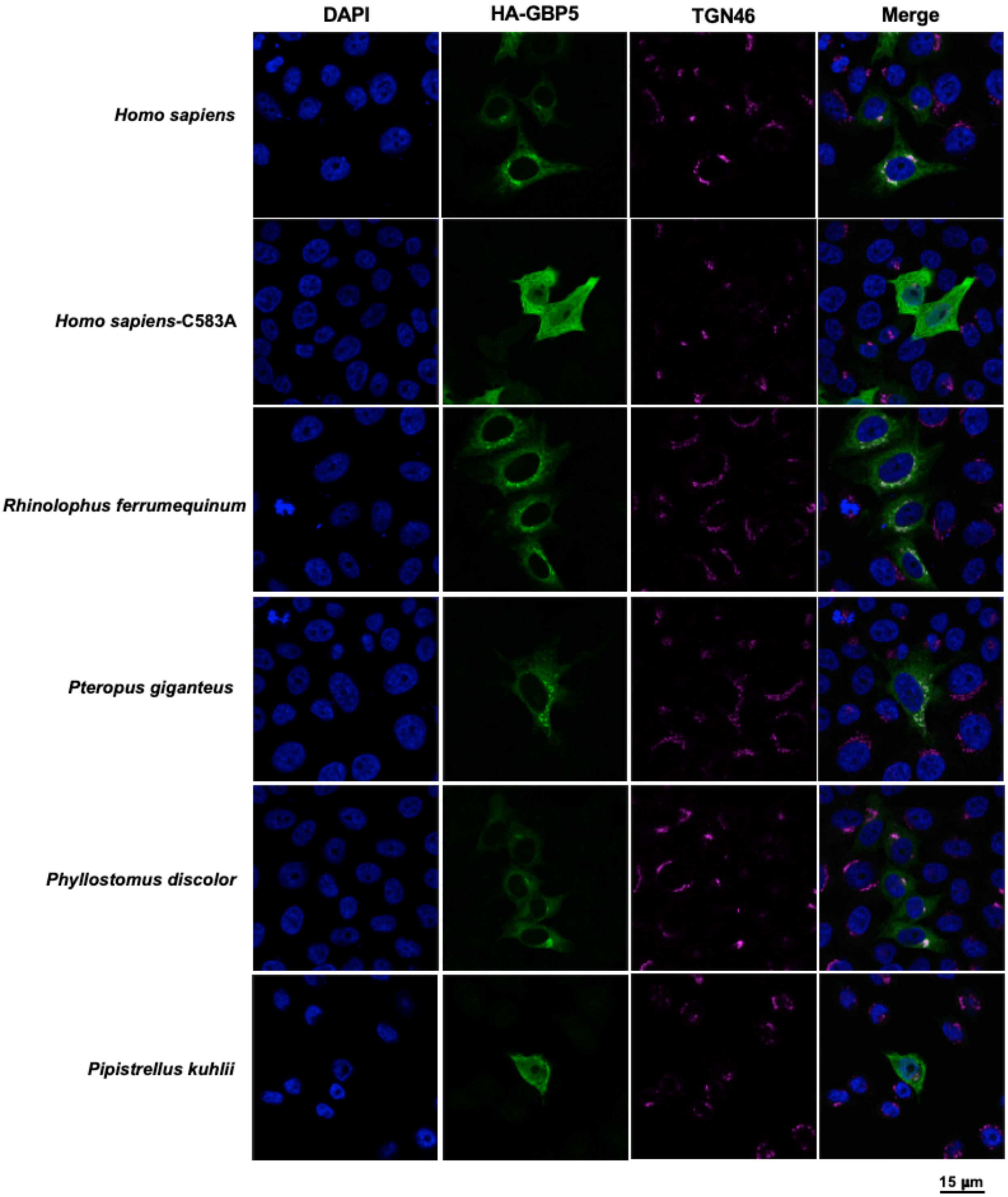

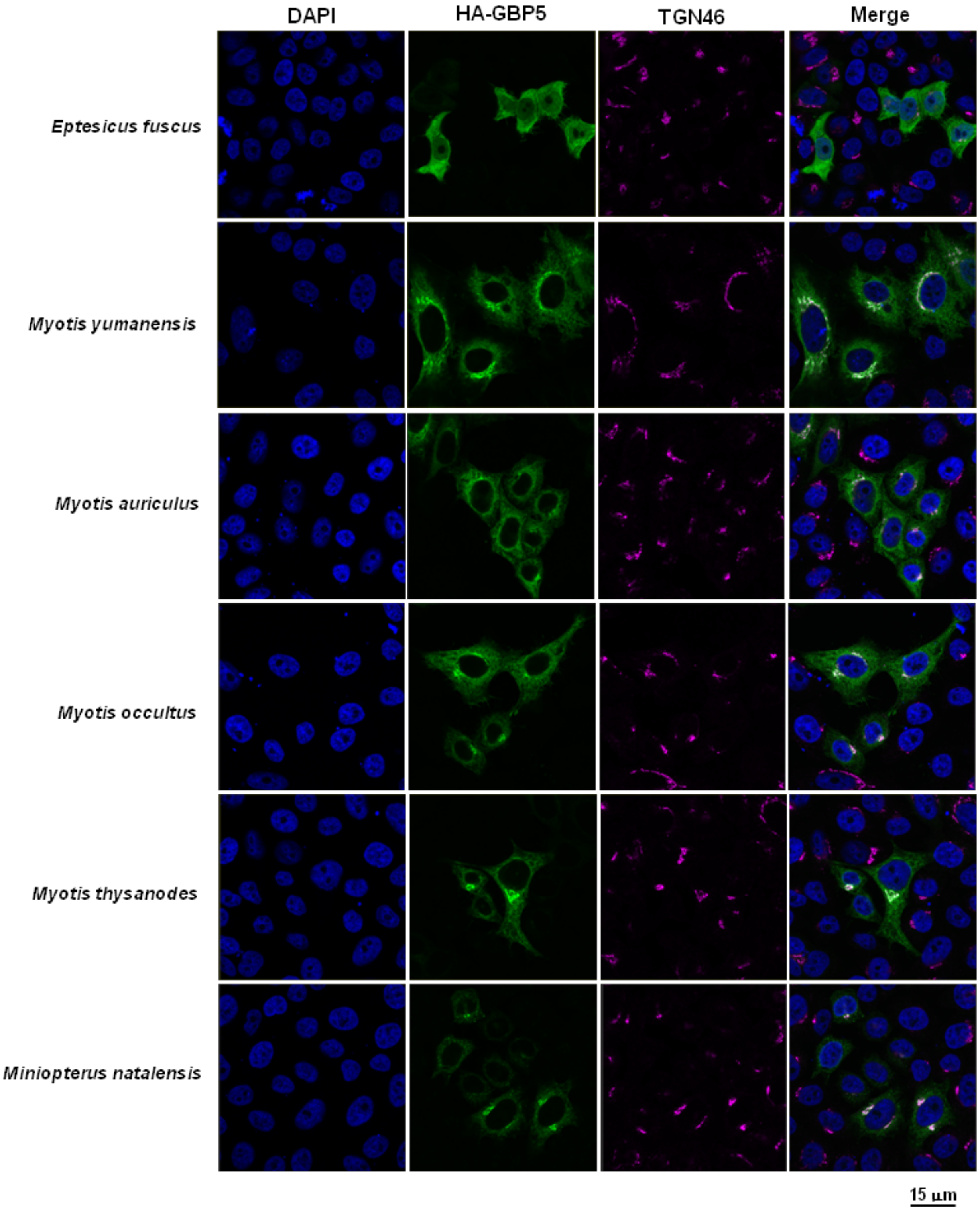
DAPI
HA-GBP5
TGN46
Merge
Eptesicus fuscus
Myotis yumanensis
Myotis auriculus
Myotis occultus
Myotis thysanodes
Miniopterus natalensis
15 µm
