# Supplementary material for: Genomic and functional adaptations in the guanylate-binding protein GBP5 highlight specificities of bat antiviral innate immunity
Source: PLoS Biol. 2026 Apr 21;24(4):e3003760. doi: 10.1371/journal.pbio.3003760 (PMC13128109; doi:10.1371/journal.pbio.3003760)

<u>Figure S4. Species-specific restriction of bat GBP5 on HIV-1 Env glycoprotein maturation and viral protein expression.</u> A, HIV-1 titers in the supernatants as quantified by RT activity (mU/ml) in the context of a dose of HA-GBP5 (1, 2 or 4 µg) or control vector (EV). The corresponding species of GBP5 is shown (name follows the UCSC nomenclature, three letters from genus followed by three letters from species). From three independent experiments, bars are SD. B, Western blot analysis of HA-GBP5, HIV-1 Env and HIV-1 Gag, and beta-actin (loading control) from the lysates of the HIV-1 producer cells (bottom) and the purified virion fraction of the supernatant (top) in the context of 4µg of the corresponding GBP5 or control vector (EV). The data underlying this Supplementary Figure can be found in Dataset S2.

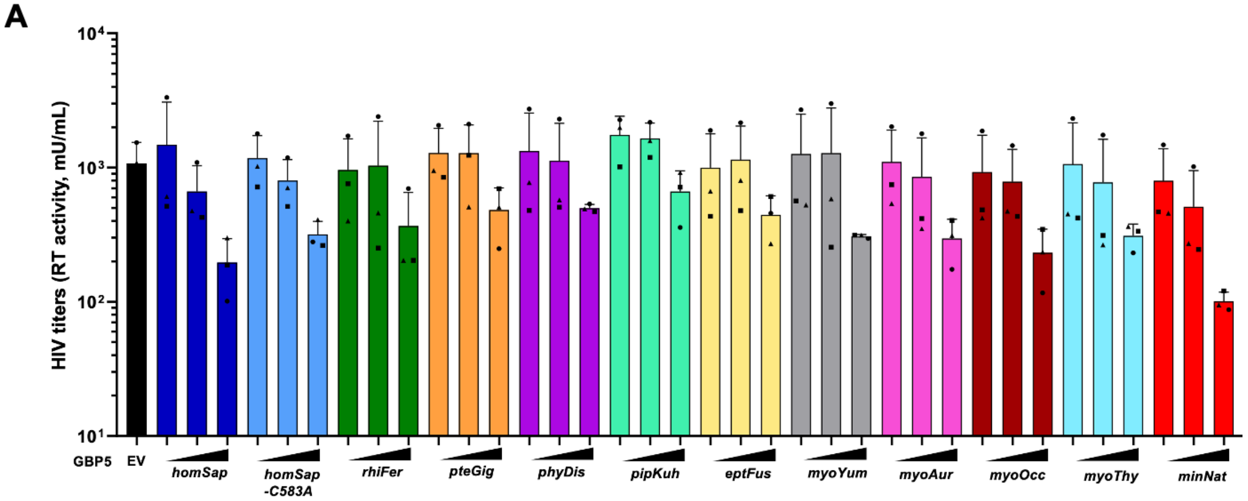

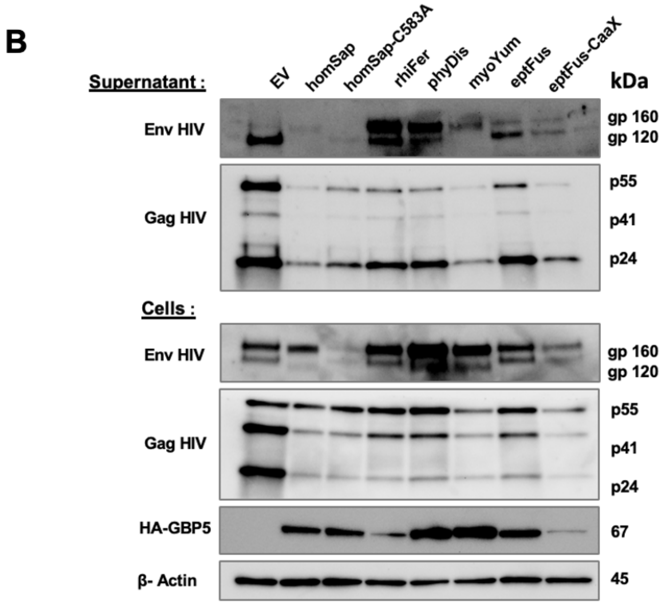

Supplement: S4 Fig — A, HIV-1 titers in the supernatants as quantified by RT activity (mU/ml) in the context of a dose of HA-GBP5 (1, 2, or 4 µg) or control vector (EV). The corresponding species of GBP5 is shown (name follows the UCSC nomenclature, three letters from genus followed by three letters from species). From three independent experiments, bars are SD. B, western blot analysis of HA-GBP5, HIV-1 Env, and HIV-1 Gag, and beta-actin (loading control) from the lysates of the HIV-1 producer cells (bottom) and the purified virion fraction of the supernatant (top) in the context of 4 µg of the corresponding GBP5 or control vector (EV). The data underlying this Supplementary Figure can be found in S2 Dataset. (PDF) [file pbio.3003760.s004.pdf]
