# Supplementary material for: Genomic and functional adaptations in the guanylate-binding protein GBP5 highlight specificities of bat antiviral innate immunity
Source: PLoS Biol. 2026 Apr 21;24(4):e3003760. doi: 10.1371/journal.pbio.3003760 (PMC13128109; doi:10.1371/journal.pbio.3003760)

Figure S5. Expression of bat GBP5 in the context of pseudoviral particle production does not increase cytotoxicity in HEK-293T cells.

A, Western.blot quantification of three independent experiments of Tubulin, as in **Fig. 5D**. B, HEK-293T cells were transfected with plasmids coding for HA-GBP5 or the empty vector (EV control), and for HIV-1 LAI genome and Luciferase reporter (Bru∂EnvLuc2 vector), NL4.3 Envelope and Rev (identical conditions used for HIV infection in **Fig. 5**). 48h post-transfection, cell viability was determined by measuring the level of adenosine triphosphate (ATP). A treatment with etoposide (100µM) during 24h was used as a positive control. Mean values of three independent experiments are shown. Statistics versus the corresponding control condition: *, *p* value <0.05. The data underlying this Supplementary Figure can be found in Dataset S2.

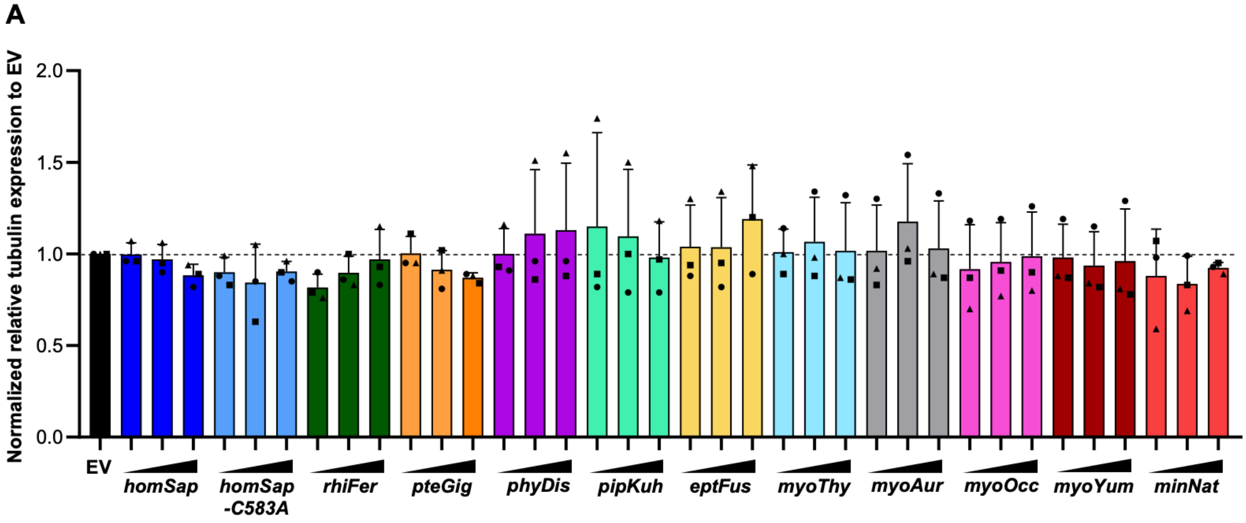

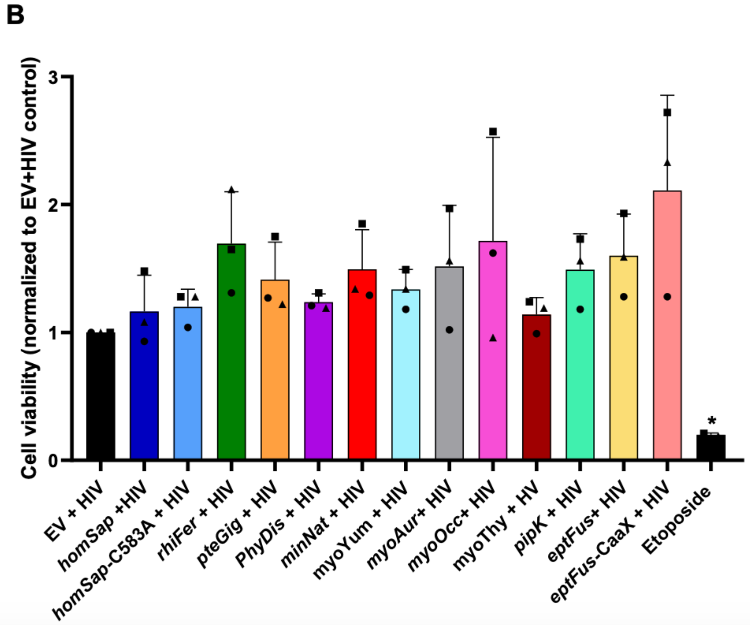

Supplement: S5 Fig — A, Western.blot quantification of three independent experiments of Tubulin, as in Fig 5D. B, HEK-293T cells were transfected with plasmids coding for HA-GBP5 or the empty vector (EV control), and for HIV-1 LAI genome and Luciferase reporter (Bru∂EnvLuc2 vector), NL4.3 Envelope and Rev (identical conditions used for HIV infection in Fig 5). 48 hours post-transfection, cell viability was determined by measuring the level of adenosine triphosphate (ATP). A treatment with etoposide (100 µM) during 24 hour was used as a positive control. Mean values of three independent experiments are shown. Statistics versus the corresponding control condition: *, p value < 0.05). The data underlying this Supplementary Figure can be found in S1 Dataset. (PDF) [file pbio.3003760.s005.pdf]
