# Supplementary material for: Genomic and functional adaptations in the guanylate-binding protein GBP5 highlight specificities of bat antiviral innate immunity
Source: PLoS Biol. 2026 Apr 21;24(4):e3003760. doi: 10.1371/journal.pbio.3003760 (PMC13128109; doi:10.1371/journal.pbio.3003760)

<u>Figure S6. Titration of pseudoviruses by RT activity in the supernatant.</u>
Titration of lentiviruses pseudotyped with EBLV-1g (A), VSVg (B), or HIV-1 Env (C) from supernatants, quantified by RT activity (mU/ml). A-B, with 2 µg of HA-GBP5 or control vector (EV) for the corresponding indicated species (i.e. HomSap, Homo sapiens). C, in the context of a dose of HA-GBP5 (1, 2 or 4 µg) or control vector (EV). EptFus-CaaX corresponds to the C-ter ancestral reconstructed *Eptesicus fuscus* GBP5 bearing the CaaX prenylation motif. Left, Normalized values to control (EV) at 1. Right, Raw data (mU/mL). The data underlying this Supplementary Figure can be found in Dataset S2.

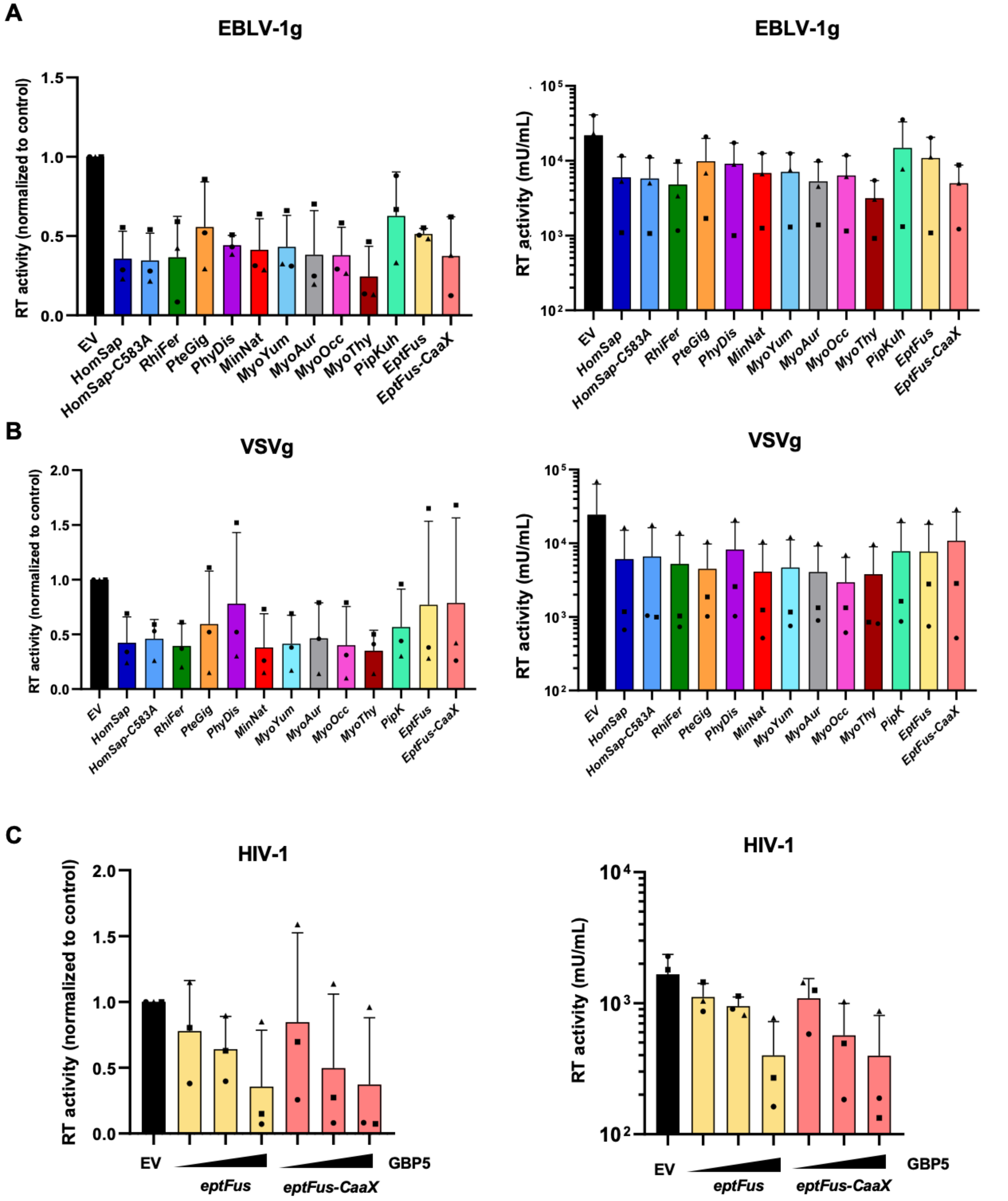

Supplement: S6 Fig — Titration of lentiviruses pseudotyped with EBLV-1g (A), VSVg (B), or HIV-1 Env (C) from supernatants, quantified by RT activity (mU/ml). A and B, with 2 µg of HA-GBP5 or control vector (EV) for the corresponding indicated species (i.e., HomSap, Homo sapiens). C, in the context of a dose of HA-GBP5 (1, 2, or 4 µg) or control vector (EV). EptFus-CaaX corresponds to the C-ter ancestral reconstructed Eptesicus fuscus GBP5 bearing the CaaX prenylation motif. Left, Normalized values to control (EV) at 1. Right, Raw data (mU/mL). The data underlying this Supplementary Figure can be found in S1 Dataset. (PDF) [file pbio.3003760.s006.pdf]
