# Supplementary material for: Genomic and functional adaptations in the guanylate-binding protein GBP5 highlight specificities of bat antiviral innate immunity
Source: PLoS Biol. 2026 Apr 21;24(4):e3003760. doi: 10.1371/journal.pbio.3003760 (PMC13128109; doi:10.1371/journal.pbio.3003760)

Figure S7. Quantification of Western-blot from independent experiments, as in Fig. 5D, testing the effect of eptFus and the CaaX mutant on viral protein expressions from Fig. 7.
The data underlying this Supplementary Figure can be found in Dataset S2.

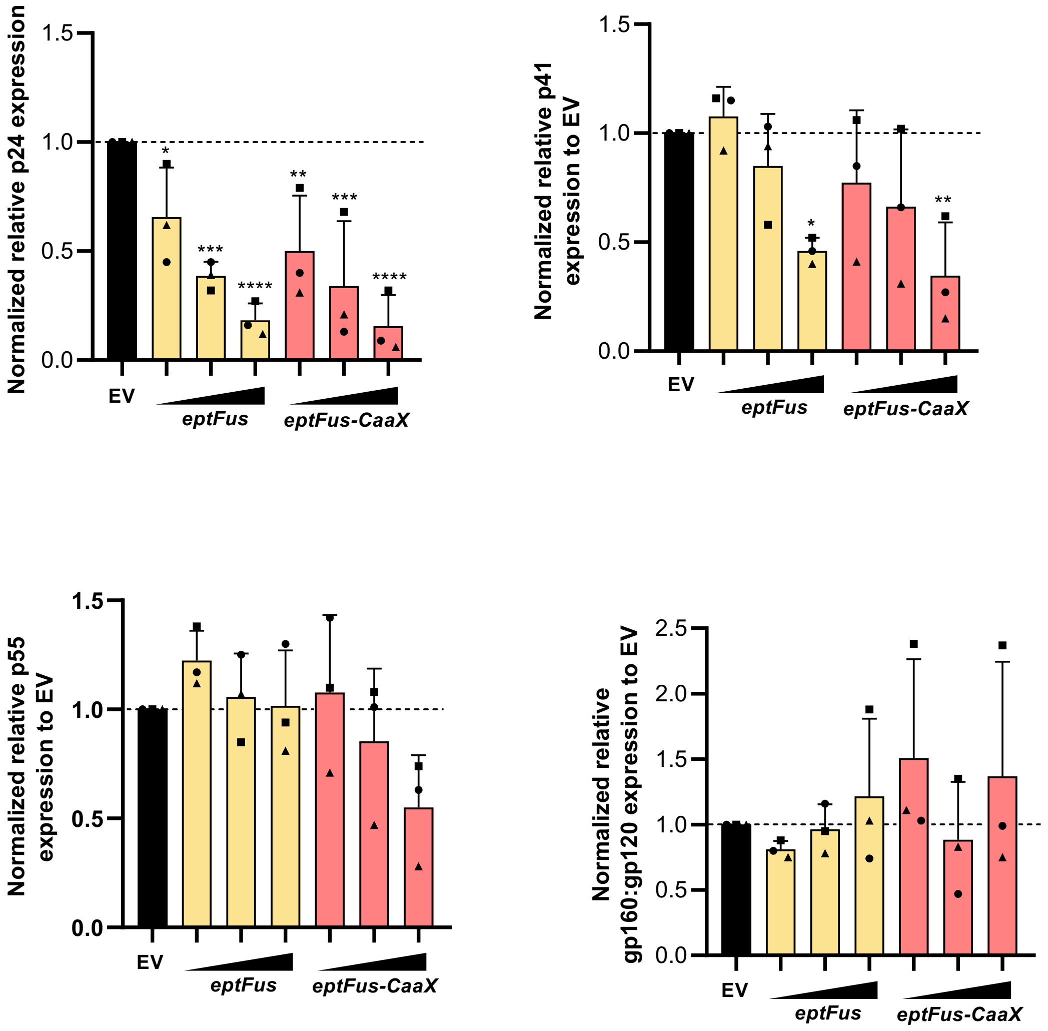

Supplement: S7 Fig — The data underlying this Supplementary Figure can be found in S2 Dataset. (PDF) [file pbio.3003760.s007.pdf]
