# Supplementary material for: Genomic and functional adaptations in the guanylate-binding protein GBP5 highlight specificities of bat antiviral innate immunity
Source: PLoS Biol. 2026 Apr 21;24(4):e3003760. doi: 10.1371/journal.pbio.3003760 (PMC13128109; doi:10.1371/journal.pbio.3003760)

Figure S8. Alignment of *Myotis yumanensis, Eptesicus fuscus* and *Homo sapiens* GBP5 protein sequences.

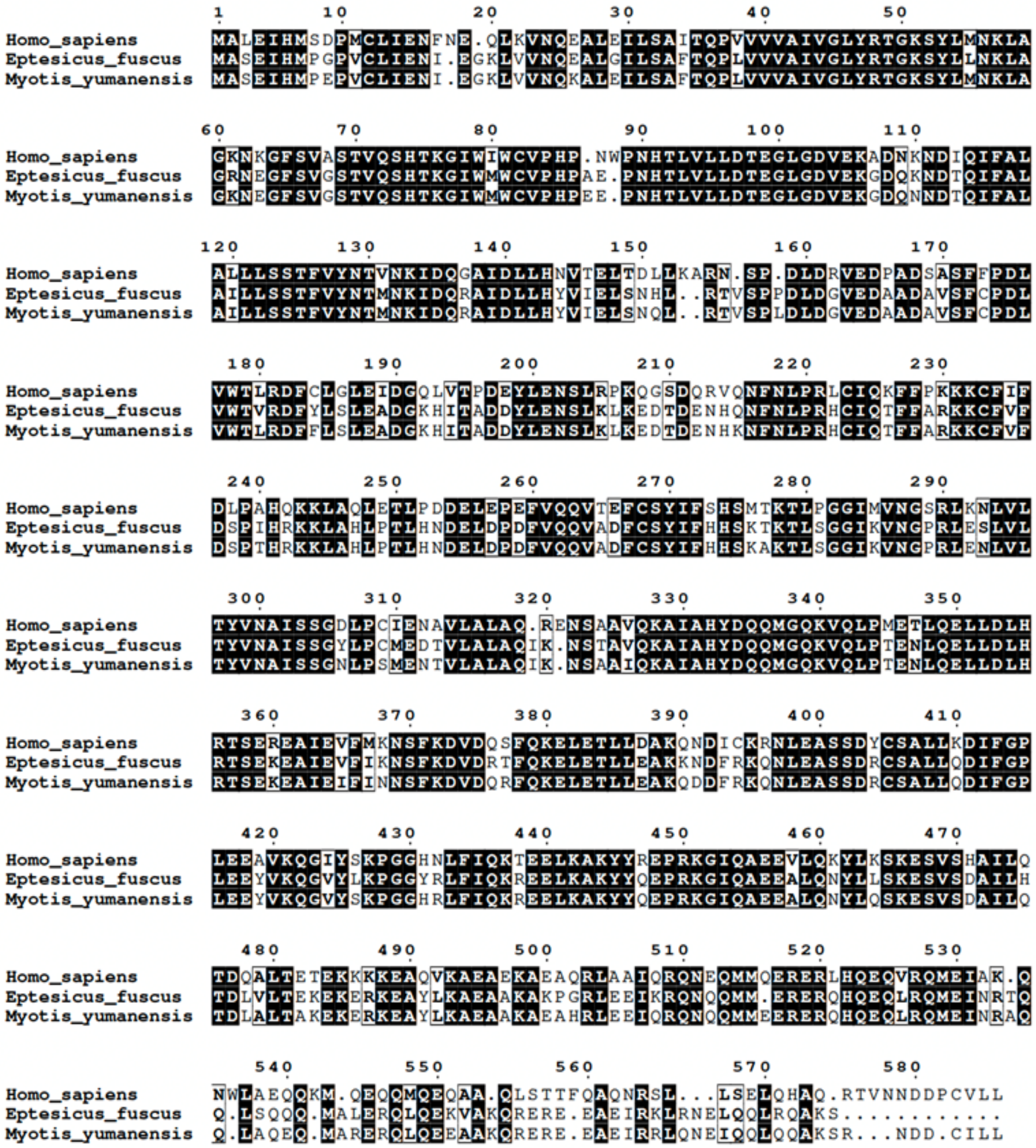

Supplement: S8 Fig — (PDF) [file pbio.3003760.s008.pdf]
