# Supplementary material for: Genomic and functional adaptations in the guanylate-binding protein GBP5 highlight specificities of bat antiviral innate immunity
Source: PLoS Biol. 2026 Apr 21;24(4):e3003760. doi: 10.1371/journal.pbio.3003760 (PMC13128109; doi:10.1371/journal.pbio.3003760)

Figure S10. Divergent residues identified between functionally tested *Myotis* GBP5s.
Of note, GBP5 from *Myotis occultus* was a stronger restrictor of the infectivity of viral particles bearing VSVg, as compared to other *Myotis* tested (Fig. 6).

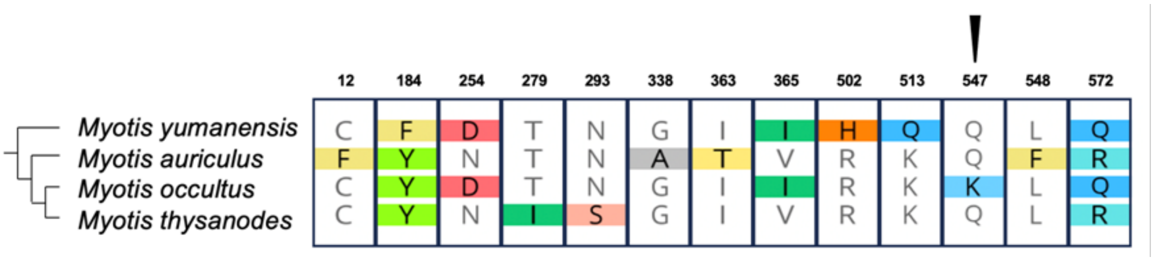

Supplement: S10 Fig — Of note, GBP5 from Myotis occultus was a stronger restrictor of the infectivity of viral particles bearing VSVg, as compared to other Myotis tested (Fig 6). (PDF) [file pbio.3003760.s010.pdf]
