# Supplementary material for: Genomic and functional adaptations in the guanylate-binding protein GBP5 highlight specificities of bat antiviral innate immunity
Source: PLoS Biol. 2026 Apr 21;24(4):e3003760. doi: 10.1371/journal.pbio.3003760 (PMC13128109; doi:10.1371/journal.pbio.3003760)

S1 Raw images. Raw immunoblots of the Figures.

A, Associated with Fig. 5; B, Associated with Fig. S4; C, Associated with Fig. 7; D, Associated with Fig. S9.

A

Figure 5

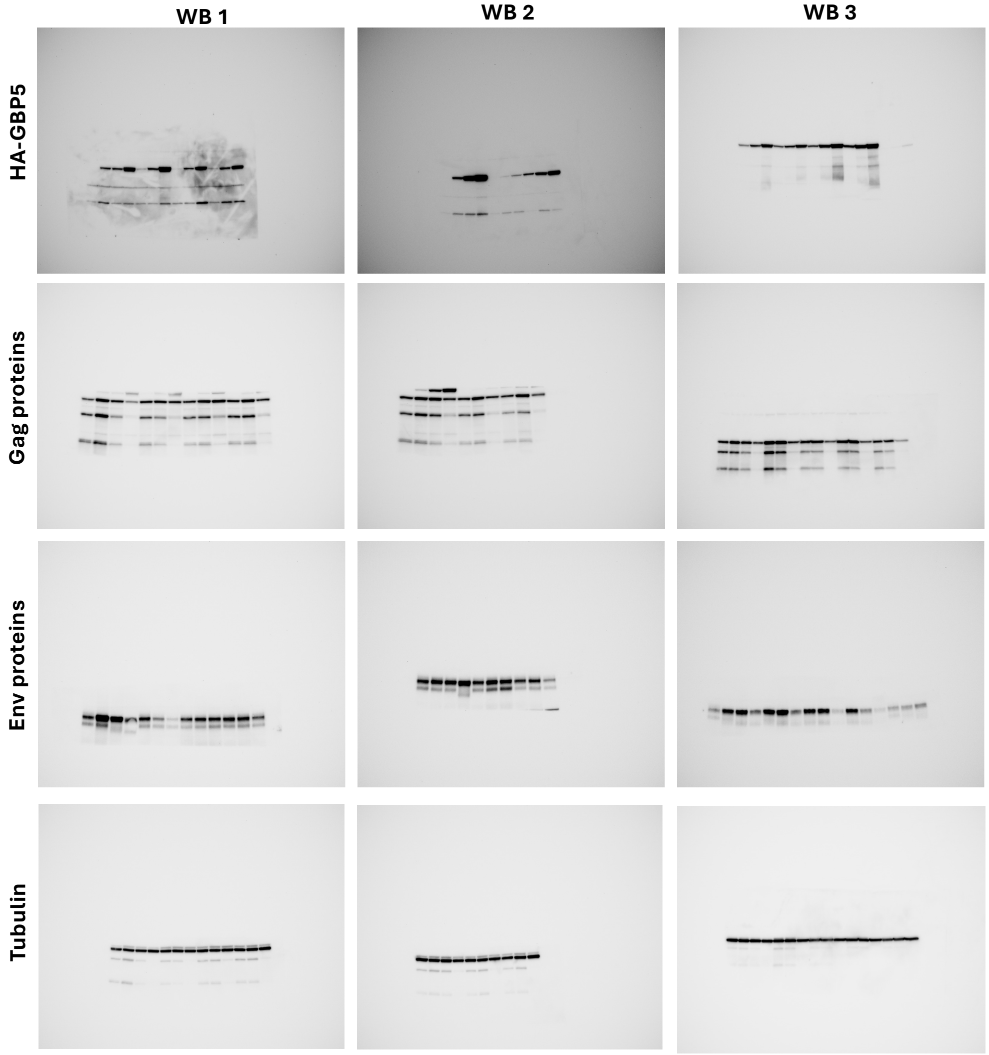

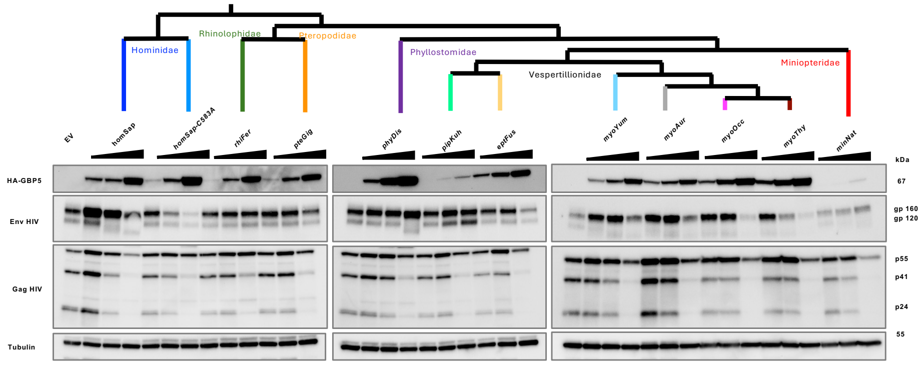

B

Figure S4

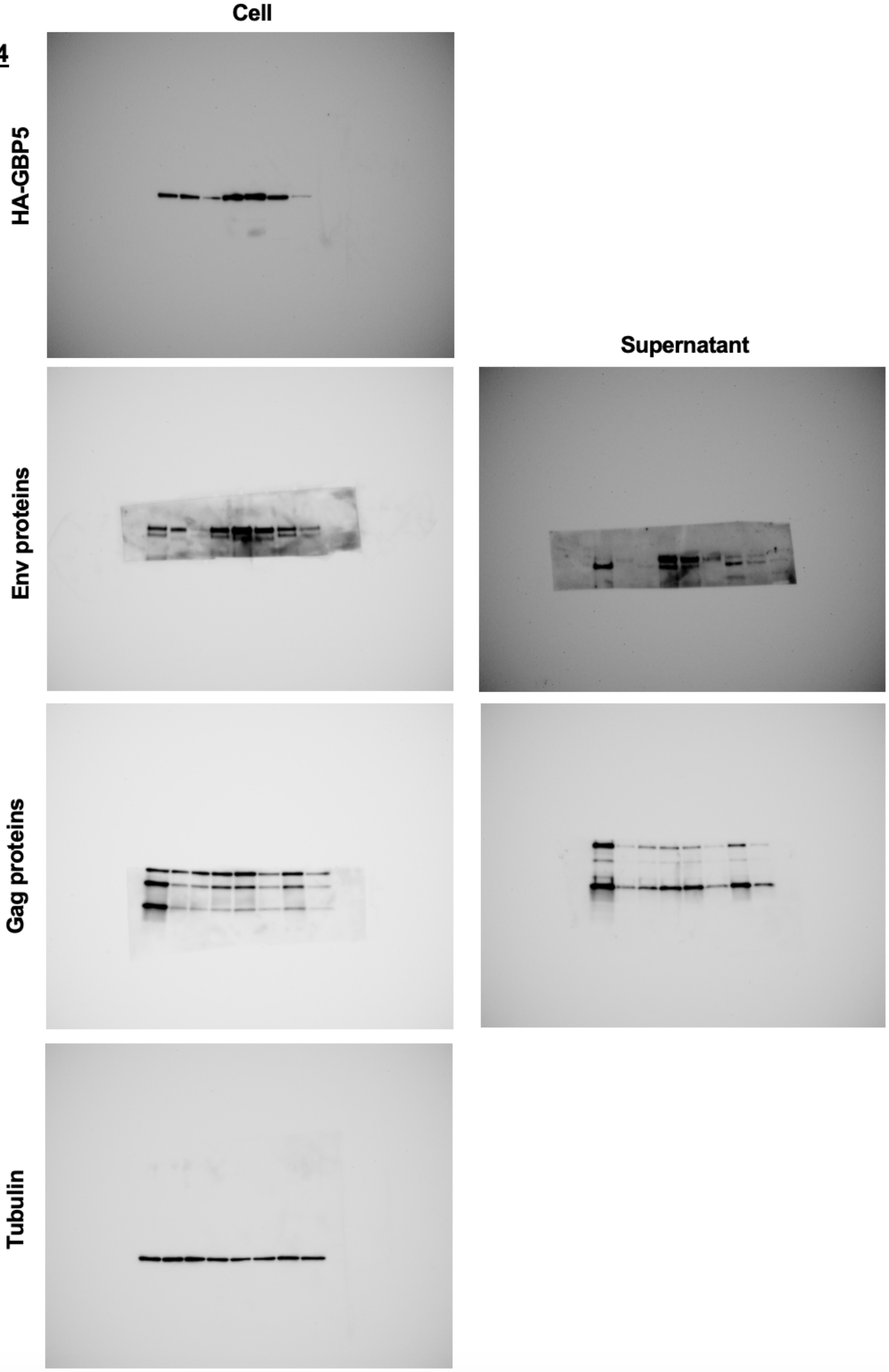
Cell
HA-GBP5
Supernatant
Env proteins
Gag proteins
Tubulin

C

Figure 7

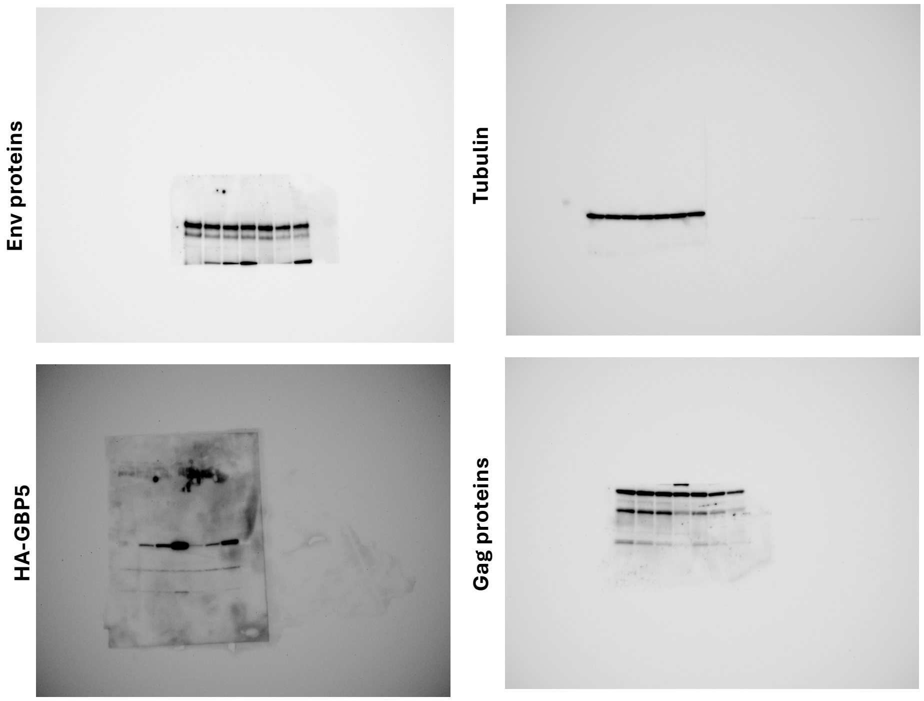
Env proteins
Tubulin
HA-GBP5
Gag proteins

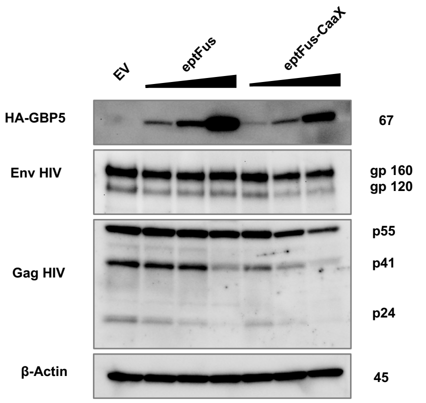
EV
eptFus
eptFus-CaaX
HA-GBP5
67
Env HIV
gp 160
gp 120
p55
Gag HIV
p41
p24
β-Actin
45

D

Figure S9

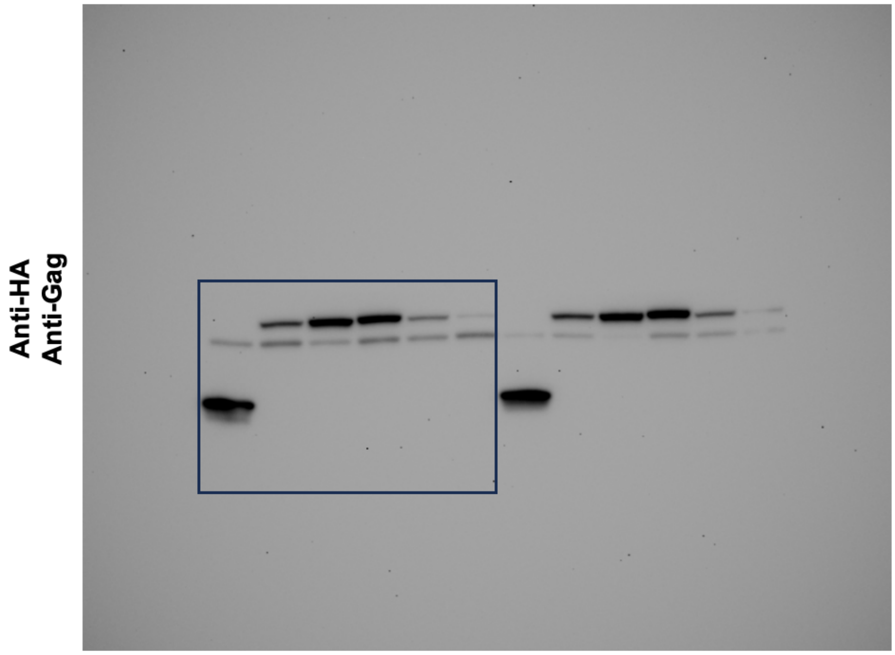

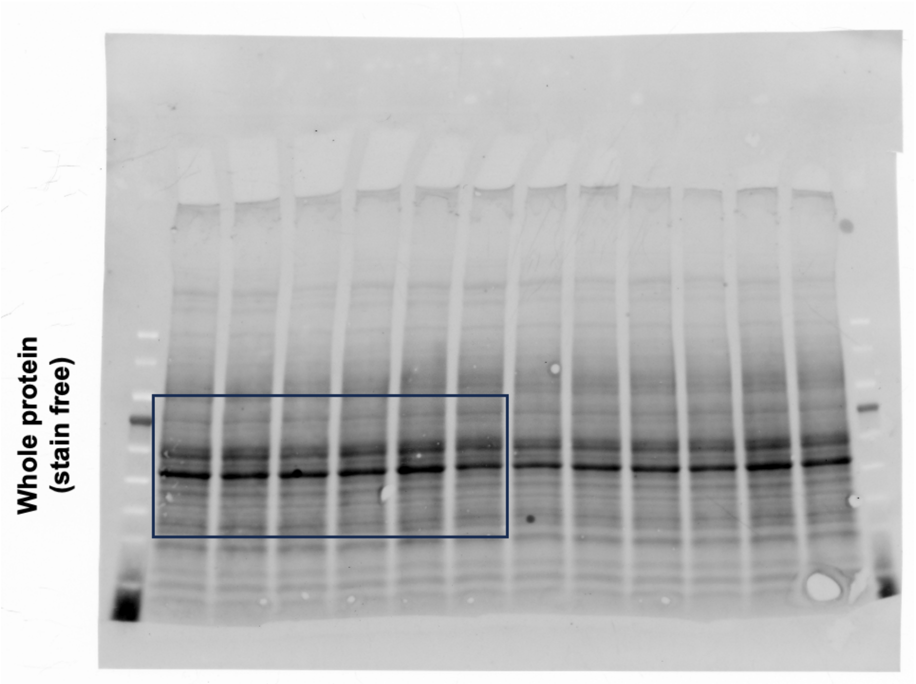

Supplement: S1 Raw Images — A, Associated with Fig 5; B, Associated with S4 Fig; C, Associated with Fig 7; D, Associated with S9 Fig. (PDF) [file pbio.3003760.s011.pdf]
