# Supplementary material for: Genomic and functional adaptations in the guanylate-binding protein GBP5 highlight specificities of bat antiviral innate immunity
Source: PLoS Biol. 2026 Apr 21;24(4):e3003760. doi: 10.1371/journal.pbio.3003760 (PMC13128109; doi:10.1371/journal.pbio.3003760)
Supplement: S1 Table — Summary of the effects of bat GBP5s on the HIV-1 intrinsic infectivity step and on RT activity in the supernatant (“viral production” in the table) relative to the EV control normalized at 100%. (PDF) [file pbio.3003760.s012.pdf]

Table S1. Bat GBP5 species-specific and dose-dependent effects on HIV-1 intrinsic infectivity (upon RT normalization) and RT activity in the supernatant.

Summary of the effects of bat GBP5s on the HIV-1 intrinsic infectivity step and on RT activity in the supernatant ("viral production" in the table) relative to the EV control normalized at 100%.

Species	GBP5 (ug)	% HIV-1 intrinsic infectivity	% viral production
EV	0	100,0	100,0
homSap	1	56,2	119,4
	2	23,7	61,9
	4	5,6	21,8
homSap-C583A	1	62,0	109,9
	2	35,8	75,9
	4	23,4	33,2
myoAur	1	73,8	106,0
	2	27,2	97,2
	4	8,2	33,4
myoOcc	1	61,7	101,7
	2	17,3	72,8
	4	4,5	35,7
myoThy	1	48,8	80,4
	2	19,4	70,1
	4	5,6	28,9
myoYum	1	69,1	87,6
	2	12,7	63,5
	4	3,3	34,8
pteGig	1	29,9	120,8
	2	34,0	129,6
	4	4,6	59,4
pipKuh	1	60,6	166,3
	2	60,3	161,8
	4	111,5	75,5
minNat	1	63,6	72,0
	2	25,1	44,0
	4	2,5	11,5
eptFus	1	82,8	85,7
	2	54,7	98,0
	4	34,4	52,0
rhiFer	1	60,0	91,7
	2	50,2	80,0
	4	14,1	32,6
phyDis	1	73,5	109,7
	2	44,6	95,6
	4	11,6	52,8
