# Supplementary material for: Genomic and functional adaptations in the guanylate-binding protein GBP5 highlight specificities of bat antiviral innate immunity
Source: PLoS Biol. 2026 Apr 21;24(4):e3003760. doi: 10.1371/journal.pbio.3003760 (PMC13128109; doi:10.1371/journal.pbio.3003760)
Supplement: S2 Table — (PDF) [file pbio.3003760.s013.pdf]

Table S2. Primer sequences.

Gene	Method	Primer name	Target	Specificity	Sequence
GBP5	PCR	vesper-GBP5-F	5'-UTR	*Vespertilionidae*	5'-CTGGAYATGGCCTCAGAGAT-3'
GBP5	PCR	vesper-GBP5-R	3'-UTR	*Vespertilionidae*	5'-CTTCAGTGYAAAGTGAACATGA-3'
GBP5	PCR	E.Fucus-GBP5-F	5'-UTR	*Eptesicus Fuscus*	5'-CGTGGACATGGCCTCAGA-3'
GBP5	Mutagenesis	Fwd_mut_EptFus_stop_Arg	CDS	*Eptesicus Fuscus*	5'-CAGCAACTACGGCAAGCAAAATCACGAAATGATGACTGTATC-3'
GBP5	Mutagenesis	Rev_mut_EptFus_stop_Arg	CDS	*Eptesicus Fuscus*	5'-GATACAGTCATCATTTCGTGATTTTGCTTGCCGTAGTTGCTG-3'
